# Current controversies in TNM for the radiological staging of rectal cancer and how to deal with them: results of a global online survey and multidisciplinary expert consensus

**DOI:** 10.1007/s00330-022-08591-z

**Published:** 2022-03-07

**Authors:** Doenja M. J. Lambregts, Nino Bogveradze, Lennart K. Blomqvist, Emmanouil Fokas, Julio Garcia-Aguilar, Bengt Glimelius, Marc J. Gollub, Tsuyoshi Konishi, Corrie A. M. Marijnen, Iris D. Nagtegaal, Per J. Nilsson, Rodrigo O. Perez, Petur Snaebjornsson, Stuart A. Taylor, Damian J. M. Tolan, Vincenzo Valentini, Nicholas P. West, Albert Wolthuis, Max J. Lahaye, Monique Maas, Geerard L. Beets, Regina G.H. Beets-Tan

**Affiliations:** 1 Department of Radiology, The Netherlands Cancer Institute, P.O. Box 90203, 1006 BE Amsterdam, The Netherlands; 2 GROW School for Oncology and Developmental Biology, Maastricht University, Maastricht, The Netherlands; 3 Department of Radiology, American Hospital Tbilisi, Tbilisi, Georgia; 4 Department of Imaging and Physiology, Karolinska University Hospital, Stockholm, Sweden; 5 Department of Radiooncology, University Hospital, Goethe University Frankfurt am Main, Frankfurt am Main, Germany; 6 Frankfurt Cancer Institute (FCI), University Hospital, Goethe University Frankfurt am Main, Frankfurt am Main, Germany; 7 Department of Surgery, Colorectal Service, Benno C. Schmidt Chair in Surgical Oncology, Memorial Sloan Kettering Cancer Center, New York, NY USA; 8 Department of Immunology, Genetics and Pathology, Uppsala University, Uppsala, Sweden; 9 Department of Radiology, Memorial Sloan Kettering Cancer Center, New York, NY USA; 10 Department of Colon and Rectal Surgery, The University of Texas MD Anderson Cancer Center, Houston, TX USA; 11 Department of Radiation Oncology, The Netherlands Cancer Institute, Amsterdam, The Netherlands; 12 Department of Radiation Oncology, Leiden University Medical Center, Leiden, The Netherlands; 13 Department of Pathology, Radboud University Medical Centre, Nijmegen, The Netherlands; 14 Department of Molecular Medicine and Surgery, Karolinska Institutet, Division of Coloproctology, Pelvic Cancer Center, Karolinska University Hospital, Stockholm, Sweden; 15 Hospital Alemão Oswaldo Cruz & Hospital Beneficência Portuguesa de São Paulo, São Paulo, Brazil; 16 Department of Pathology, The Netherlands Cancer Institute, Amsterdam, The Netherlands; 17 Centre for Medical Imaging, University College London Hospital, London, UK; 18 Department of Radiology, Leeds Teaching Hospitals NHS Trust, Leeds, UK; 19 Department of Bioimaging, Radiation Oncology and Hematology, Fondazione Policlinico Universitario “A. Gemelli” IRCCS, Università Cattolica S. Cuore, Rome, Italy; 20 Pathology & Data Analytics, Leeds Institute of Medical Research at St James’s, University of Leeds, Leeds, UK; 21 Department of Abdominal Surgery, University Hospitals Leuven, Leuven, Belgium; 22 Department of Surgery, The Netherlands Cancer Institute, Amsterdam, The Netherlands; 23 Institute of Regional Health Research, University of Southern Denmark, Odense, Denmark

**Keywords:** Rectal neoplasms, Neoplasm staging, Magnetic resonance imaging, Consensus, Guideline

## Abstract

**Objectives:**

To identify the main problem areas in the applicability of the current TNM staging system (8^th^ ed.) for the radiological staging and reporting of rectal cancer and provide practice recommendations on how to handle them.

**Methods:**

A global case-based online survey was conducted including 41 image-based rectal cancer cases focusing on various items included in the TNM system. Cases reaching < 80% agreement among survey respondents were identified as problem areas and discussed among an international expert panel, including 5 radiologists, 6 colorectal surgeons, 4 radiation oncologists, and 3 pathologists.

**Results:**

Three hundred twenty-one respondents (from 32 countries) completed the survey. Sixteen problem areas were identified, related to cT staging in low-rectal cancers, definitions for cT4b and cM1a disease, definitions for mesorectal fascia (MRF) involvement, evaluation of lymph nodes versus tumor deposits, and staging of lateral lymph nodes. The expert panel recommended strategies on how to handle these, including advice on cT-stage categorization in case of involvement of different layers of the anal canal, specifications on which structures to include in the definition of cT4b disease, how to define MRF involvement by the primary tumor and other tumor-bearing structures, how to differentiate and report lymph nodes and tumor deposits on MRI, and how to anatomically localize and stage lateral lymph nodes.

**Conclusions:**

The recommendations derived from this global survey and expert panel discussion may serve as a practice guide and support tool for radiologists (and other clinicians) involved in the staging of rectal cancer and may contribute to improved consistency in radiological staging and reporting.

**Key Points:**

• *Via a case-based online survey (incl. 321 respondents from 32 countries), we identified 16 problem areas related to the applicability of the TNM staging system for the radiological staging and reporting of rectal cancer.*

• *A multidisciplinary panel of experts recommended strategies on how to handle these problem areas, including advice on cT-stage categorization in case of involvement of different layers of the anal canal, specifications on which structures to include in the definition of cT4b disease, how to define mesorectal fascia involvement by the primary tumor and other tumor-bearing structures, how to differentiate and report lymph nodes and tumor deposits on MRI, and how to anatomically localize and stage lateral lymph nodes.*

• *These recommendations may serve as a practice guide and support tool for radiologists (and other clinicians) involved in the staging of rectal cancer and may contribute to improved consistency in radiological staging and reporting.*

**Supplementary Information:**

The online version contains supplementary material available at 10.1007/s00330-022-08591-z.

## Introduction

The “tumor node metastasis” (TNM) system is the most applied staging system in oncology. Although not specifically designed for radiological staging, TNM has been widely adopted by radiologists for diagnostic reporting of cancer, including rectal cancer. Still, there are several controversies in the radiological application of the TNM system for rectal cancer, leading to heterogeneity in reporting [1].

This study aims to gain further insight into these controversies and identify the main problem areas in using the current TNM (8^th^ ed.) for the radiological reporting of rectal cancer. To this end, a global online survey with an emphasis on MRI for local staging was undertaken. This paper reports the outcome of this survey and the recommendations from a multidisciplinary expert panel on how to address the identified problem areas.

## Methods

**Fig. 1:**
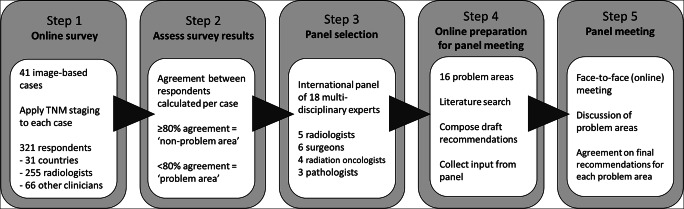
Study outline

This study included five main steps (Fig. 1):

### 1—Online survey

An online survey (using Google forms) was constructed by two of the organizing authors (D.M.J.L., N.B.) including 41 cases/questions covering the main staging items included in TNM8 [2]. Every case included a single MRI (or CT) image and schematic representation and description of the imaging findings. Respondents were asked to answer each question based on these pre-specified imaging findings without having to interpret the images themselves. Cases were organized in 6 sections focused on clinical T staging (cT), anal canal involvement, mesorectal fascia (MRF) involvement, lymph nodes and tumor deposits, regional versus non-regional lymph nodes, and M staging. Respondents were also asked some general questions about their background and use of TNM in their clinical practice. The survey was distributed via the organizing authors’ professional networks and via member mailings of various (inter)national radiological and clinical societies. The full survey is provided in Supplement 1.

### 2—Analysis of survey results

Two of the organizing authors (D.M.J.L., N.B.) analyzed the survey results and calculated for each case/question the percentage agreement between respondents. Cases reaching ≥ 80% agreement were classified as “non-problem” areas; cases reaching < 80% agreement were classified as problem areas.

### 3—Panel selection

An international expert panel was composed, including five radiologists (L.K.B., M.J.G., S.A.T., D.J.M.T., R.G.H.B-T.), six colorectal surgeons (J.G-A., T.K., P.J.N., R.O.P., A.W., G.L.B.), four radiation oncologists (E.F., B.G., C.A.M., V.V.), and three pathologists (I.D.N., P.S., N.P.W.), each with recognized expertise in the field.

### 4—Preparation for panel meeting

Two of the organizing authors (D.M.J.L., N.B.) performed a focused review of the available literature related to the identified problem areas. For each problem area, a draft recommendation (when feasible) was constructed. These were sent to all panelists to acquire their input prior to the face-to-face meeting. Panelists could indicate whether they agreed with the proposed recommendation and provide their comments and suggestions.

### 5—Panel meeting

The face-to-face panel meeting took place online on June 1, 2021; 15/18 panelists attended. Each problem area (+ input acquired in step 4) was discussed and voted on. This process was repeated until a single recommendation was decided on. Two non-voting observers (D.M.J.L., N.B.) documented key discussion points and outcomes of the voting rounds. The three panelists who did not attend approved the documented recommendations afterwards via email.

## Results

### Respondents

The survey was completed by 321 respondents (from 32 countries), including 255 radiologists and 66 other clinicians. Further details are provided in Table 1. TNM8 was routinely used by 63% of respondents; 25% used previous TNM editions and 13% did not use TNM or did not know which TNM edition was being used in their center.

**Table 1:** Main characteristics of the survey respondents

	No. of participants	%
**Total**	321	100%
**Country of residence**		
Netherlands	86	27%
UK	51	16%
USA	24	8%
Portugal	17	5%
Australia	15	5%
India	14	4%
Sweden	13	4%
Italy	12	4%
Brazil	11	3%
Other (< 10 per country)*	78	24%
**Profession**		
Radiologist	255	79%
Abdominal radiologist with specific expertise in rectal MRI	103	32%
Abdominal radiologist	87	27%
General radiologist	39	12%
Resident	26	8%
Surgeon	34	11%
Radiation oncologist	16	5%
Pathologist	6	2%
Other**	10	3%
**TNM staging applied in clinical practice**		
TNM 8	201	63%
TNM 7	77	24%
Older version (TNM 6 or older)	2	1%
None	10	3%
Unknown	31	10%

*Other countries with < 10 respondents included Argentina, Belgium, Bulgaria, Canada, China, Denmark, France, Georgia, Germany, Greece, Ireland, Israel, Korea, New Zealand, Norway, Poland, Romania, Scotland, Serbia, Slovenia, Spain, Switzerland, Ukraine

**Other professions included medical oncologist (*n* = 7), gastroenterologist (*n* = 2), and PhD researcher (*n* = 1)

### Survey outcomes

Detailed survey outcomes are provided in Table 2. Respondents reached ≥ 80% agreement for 25/41 (61%) of cases. The remaining 16 (39%) were classified as problem areas, related to:

- cT staging in anal canal involvement
- Definitions for cT4b disease
- cT staging in MRF vs. peritoneal involvement
- Definitions for MRF involvement
- Definitions for lymph nodes versus tumor deposits
- Definitions to assess regional and non-regional lymph nodes
- Definitions for M1a disease

**Table 2:** Survey results

Section 1—cT staging* Respondents were asked to assign a cT stage for each case	**% consensus**	
Case 01: tumor limited to the bowel wall (i.e., cT1–2)	100%	cT1-2
Case 02: tumor penetrating the wall and extending into perirectal fat, wide margin between tumor and MRF (i.e., cT3)	98%	cT3
Case 03: tumor invading the seminal vesicles and prostate (i.e., cT4b)	97%	cT4b
**Case 04: tumor extending into the perirectal fat, invading the MRF (i.e., cT3)**	**75%**	**cT3**
Case 05: tumor extending into the perirectal fat, invading the anterior peritoneal reflection (i.e., cT4a)	94%	cT4a
Case 06: tumor extending into the perirectal fat, invading the peritoneum above the peritoneal reflection (i.e., cT4a)	89%	cT4a
**Case 07: tumor extending beyond the MRF into the obturator space (without vessel or muscle invasion)**	**57%**	**cT3**
Section 2—anal sphincter and pelvic floor invasion* Respondents were asked to assign a cT stage for each case	**% consensus**	
**Case 08: tumor invading the internal anal sphincter**	**45%**	**cT1-2**
**Case 09: tumor invading the intersphincteric plane**	**68%**	**cT3**
**Case 10: Tumor invading the external anal sphincter**	**51%**	**cT4b**
**Case 11: Tumor invading the pelvic floor (levator ani)**	**73%**	**cT4b**
**Section 3—mesorectal fascia (MRF) involvement** Respondents were asked to determine for each case whether the MRF was involved (MRF+) or not involved (MRF−)	**% consensus**	
Case 12: tumor extending into perirectal fat (below peritoneal reflection), distance of 0 mm between tumor and MRF (i.e., MRF+)	96%	MRF+
**Case 13: tumor extending into perirectal fat (below peritoneal reflection), distance of < 1 mm between tumor and MRF (i.e., MRF+)**	**79%**	**MRF+**
**Case 14: tumor extending into perirectal fat (below peritoneal reflection), distance of 1–2 mm between tumor and MRF**	**79%**	**MRF-**
**Case 15: tumor extending into perirectal fat anteriorly (above peritoneal reflection), invading the peritoneum (i.e., MRF−)**	**51%**	**MRF-**
Case 16: tumor extending into perirectal fat posteriorly (above peritoneal reflection), distance of 0 mm between tumor and MRF (i.e., MRF+)	86%	MRF+
**Case 17: N+ lymph node** ***without*** **extracapsular extension directly adjacent to MRF**	**57%**	**MRF-**
Case 18: N+ lymph node with extracapsular extension directly adjacent to MRF	85%	MRF+
**Section 4—Nodal staging** For case 19–21, respondents were asked to classify each shown lesion as a lymph node or deposit For case 22–27, respondents were asked to assign a cN stage (cN1a, cN1b, cN1c, cN2a, cN2b) for each case	**% consensus**	
Case 19: nodular lesion in mesorectum	89%	node
Case 20: irregular mass in mesorectum	84%	deposit
**Case 21: partly nodular, partly irregular mass in mesorectum**	**43%**	**node**
Case 22: single metastatic node in mesorectum (i.e., cN1a)	98%	cN1a
Case 23: two metastatic nodes in mesorectum (i.e., cN1b)	94%	cN1b
Case 24: single tumor deposit in mesorectum (no additional nodes) (i.e., cN1c)	92%	cN1c
**Case 25: single tumor deposit plus single metastatic node in mesorectum**	**52%**	**cN1c**
Case 26: seven metastatic lymph nodes in mesorectum (i.e., cN2b)	95%	cN2b
Case 27: four metastatic lymph nodes in mesorectum (i.e., cN2a)	94%	cN2a
**Section 5—regional versus non-regional lymph nodes** Respondents were asked to determine whether lymph nodes were regional (N) or non-regional (M)	**% consensus**	
Case 28: mesorectal lymph node (i.e., regional)	100%	regional
**Case 29: obturator lymph node (i.e., regional)**	**58%**	**regional**
Case 30: external iliac lymph node (i.e., non-regional)	80%	non-regional
**Case 31: internal iliac lymph node (i.e., regional)**	**67%**	**regional**
Case 32: common iliac lymph node (i.e., non-regional)	85%	non-regional
**Case 33: inguinal node in distal tumor extending below dentate line (i.e., regional)**	**51%**	**non-regional**
Case 34: inguinal node in mid-rectal **tumor** not extending into the anal canal (i.e., non-regional)	96%	non-regional
**Section 6—M staging** Respondents were asked to assign a cM stage (cM1a, cM1b, cM1c)	**% consensus**	
Case 35: common iliac lymph node metastasis (i.e., cM1a)	94%	cM1a
Case 36: liver + para-aortic lymph node metastases (i.e., cM1b)	94%	cM1b
Case 37: unilateral lung metastases (right lung) (i.e., cM1a)	84%	cM1a
**Case 38: bilateral lung metastases (right + left lung) (i.e., cM1a)**	**56%**	**cM1b**
Case 39: liver + renal + spleen metastases (i.e., cM1b)	86%	cM1b
Case 40: peritoneal metastases (i.e., cM1c)	97%	cM1c
Case 41: peritoneal + liver metastases (i.e., cM1c)	97%	cM1c

Note, cases that did not reach ≥ 80% consensus among survey respondents are printed in bold and were defined as “problem areas”

*In cases related to cT staging, the answer options cT1, cT2, and cT12 (unable to differentiate between cT1 and cT2) were grouped together for calculation of agreement. In all other cases, agreement was calculated based on individual answer options.

Specified subgroup results (per profession and experience level) are provided in Supplement 2. In 4 out of 16 problem cases, borderline agreement (73–79%) was reached, with ≥ 80% agreement for the subgroups of MRI experts and/or abdominal radiologists.

### Panel recommendations

**Fig. 2:**
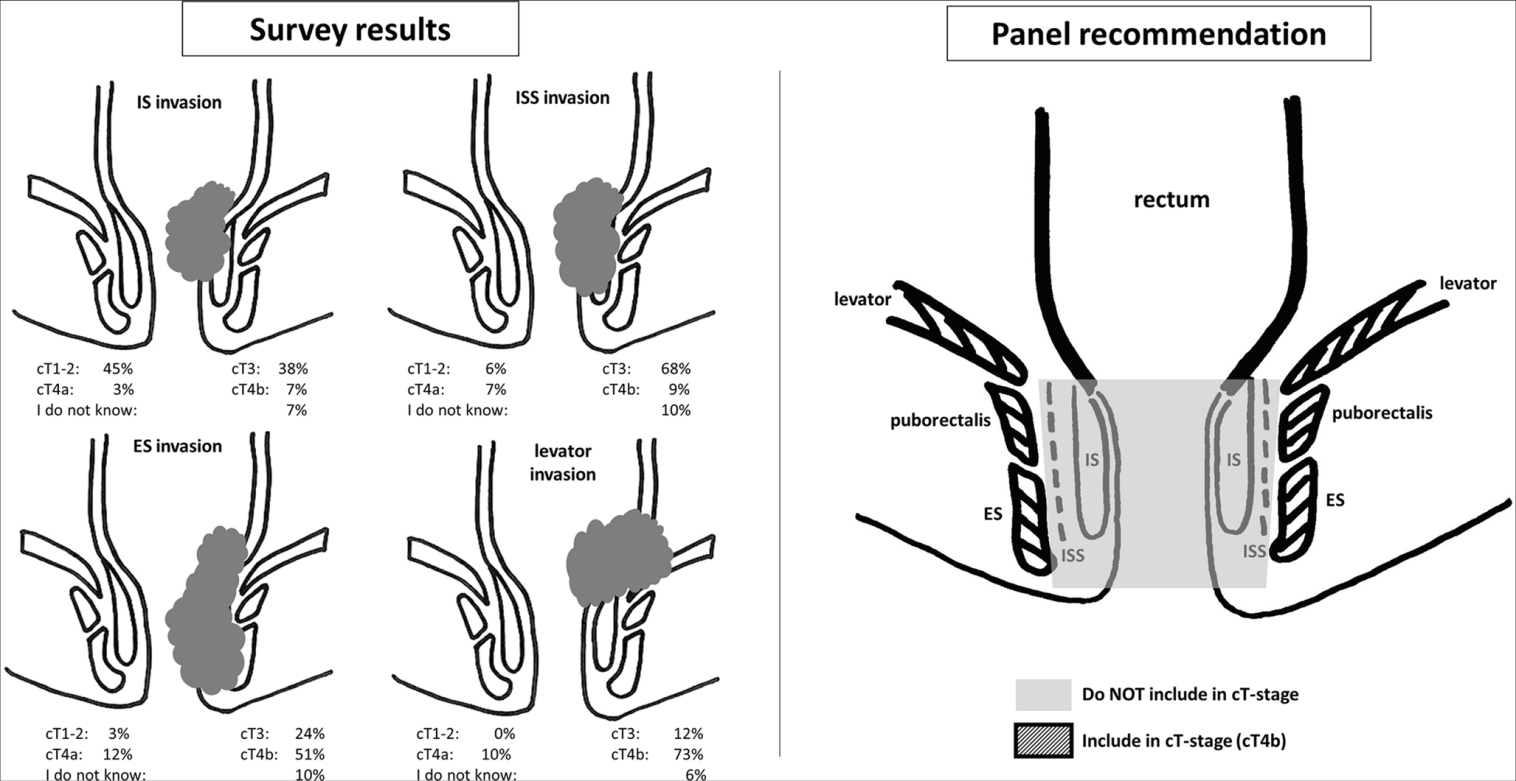
Left: survey results showing substantial variation in assessment of cT staging in cases with various degrees of anal sphincter or pelvic floor invasion. Right: panel recommendations stating not to include the internal sphincter (IS) and intersphincteric space (ISS) in cT-stage categorization, and to consider invasion of external sphincter (ES), puborectalis, and levator ani muscles (i.e., skeletal muscles) as cT4b disease

**Fig. 3:**
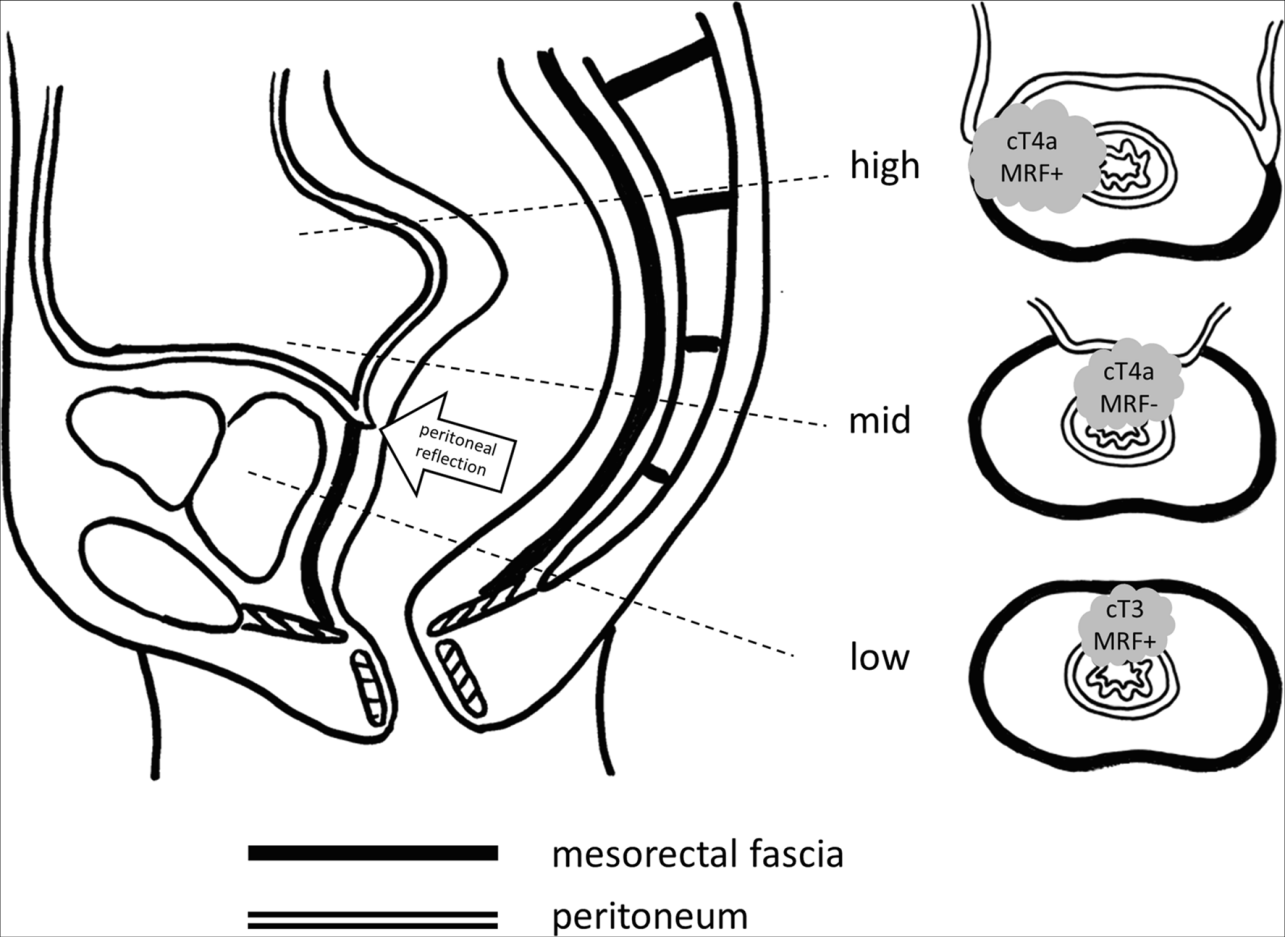
Anatomical overview of the lining of the mesorectal compartment by the MRF and peritoneum in the low, middle, and high parts of the rectum. Above the anterior peritoneal reflection, the mesorectum is lined by peritoneum anteriorly (mid) and anterolaterally (high). The remaining mesorectum is lined by the MRF. Invasion of the MRF constitutes cT3 MRF+ disease, while invasion of the peritoneum or peritoneal reflection constitutes cT4a disease. When both the peritoneum and MRF are involved, this constitutes cT4a MRF+ disease

**Fig. 4:**
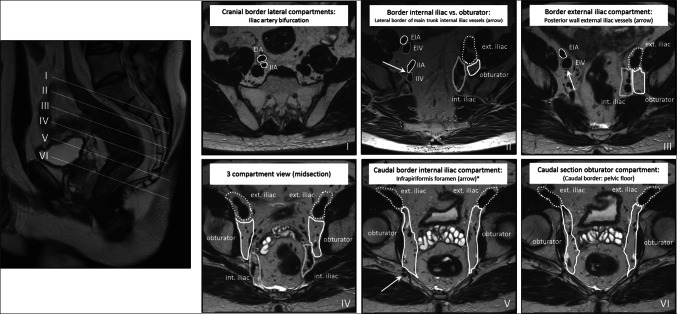
Anatomical boundaries of lateral lymph node stations (external iliac, internal iliac, obturator) on MRI. EIA = external iliac artery, EIV = external iliac vein, IIA = internal iliac artery, IIV = internal iliac vein. The border between the internal iliac and obturator compartments is defined by the lateral border of the main trunk of the internal iliac vessels (II–IV). The posterior wall of the EIV defines the border between the external iliac and obturator plus internal iliac compartments (II–VI). *The infrapiriformis foramen represents the transit point of the internal iliac vessels from the internal iliac compartment into the pudendal canal (V). This figure is largely based on a map previously published by Ogura et al JAMA Surg 2019;254: e192172 (supplement) [26]

The panel recommendations for each problem area are detailed in Table 3. Figures 2 and 3 illustrate recommendations on cT staging in low-rectal cancers, and for MRF versus peritoneal involvement. Figure 4 provides an anatomical MRI map for lateral lymph node stations.

**Table 3:** Problem areas and expert panel recommendations

**Problem area**	**Recommendation**
**cT staging**	
How to categorize cT stage in low-rectal cancers involving the anal canal or pelvic floor?	**See also Fig.** **2** • cT stage should be defined primarily based on the extent of tumor invasion at the level of the rectum. • Involvement of the internal sphincter and intersphincteric plane should *not* be taken into account when classifying the cT stage. • Involvement of the external sphincter, puborectalis, and/or levator ani muscles should be categorized as cT4b disease (=skeletal muscle invasion). • Separate from cT-stage categorization, in any low-rectal tumor, a rectal MRI report should include a detailed prose description of whether and to what extent the tumor invades the different anatomical layers of the anal sphincter and/or pelvic floor. Any involvement of the anal canal should also be routinely included in the conclusion of the report, preferably as a suffix. For example cT… (anal+), or cT… (anal−) when there is no involvement. • Note, in order to properly assess involvement of the anal canal, availability of a good-quality high-resolution coronal T2-weighted imaging sequence planned parallel to the anal canal is paramount.
How to categorize cT stage in case of mesorectal fascia (MRF) involvement and/or involvement of the peritoneum or peritoneal reflection?	**See also Fig.** **3** • Below the anterior peritoneal reflection, the mesorectum is covered by the MRF circumferentially. The MRF is not a synonym for peritoneum, and involvement of (but not beyond) the MRF should be classified as cT3 MRF+ disease. • At and above the level of the anterior peritoneal reflection, the mesorectum is partly covered by peritoneum anteriorly (mid rectum) and anterolaterally (high rectum). When the peritoneum (or peritoneal reflection) is invaded, this constitutes cT4a disease and the MRF should *not* be classified as involved, except when there is simultaneous invasion of the MRF (laterally or dorsally) in which case MRF involvement should be reported separately (i.e., as cT4a MRF+).
Definition of cT4b disease	• cT4b includes invasion of: - pelvic organs including uterus, ovaries, vagina, prostate, seminal vesicles, bladder, ureters, urethra, bone - skeletal/striated muscle (incl. obturator, piriformis, ischiococcygeus, levator ani, puborectalis, and external anal sphincter) - sciatic or sacral nerves - sacrospinous/sacrotuberous ligaments - any vessel outside the mesorectal compartment - any loop of small or large bowel in the pelvis (separate from the primary site from which the tumor originates) - any extramesorectal fat in an anatomical compartment of the pelvis outside the mesorectum, i.e., beyond the mesorectal fascia (obturator, para-iliac, or ischiorectal) • *Excluded* from cT4b are: - The mesorectal fascia (=cT3 MRF+) - The peritoneum including the anterior peritoneal reflection (=cT4a) - The internal anal sphincter and intersphincteric space (=cT1/2/3 anal+)
**Mesorectal fascia involvement**	
Which distance between tumor and MRF defines an “involved” MRF and should we consider the sub-category of a “threatened” MRF?	• Direct invasion of the MRF by the primary tumor or a margin of ≤ 1 mm between the primary tumor and MRF should be considered MRF+ (involved MRF). • The definition of a “threatened” MRF (1–2 mm) should be discarded.
How to stage the MRF in case of tumor-bearing structures (lymph nodes, deposits, EMVI) other than the primary tumor involving the MRF?	• MRF should be considered as *involved* (MRF+) in case of direct invasion or a margin of ≤ 1 mm from: - primary tumor - EMVI - tumor deposits or irregular pathologic nodes (i.e. nodes with extracapsular extension) • MRF should be considered as *non-involved* (MRF−) in case of a margin of ≤ 1 mm from: - Enlarged lymph nodes without any signs of extracapsular extension (i.e. smooth enlarged nodes) • In cases with an involved MRF, it is useful to include a suffix in the conclusion of the radiology report, describing whether the cause of involvement was the primary tumor or another structure, e.g., “MRF+ (primary)” of “MRF+ (non-primary).”
**Lymph nodes and tumor deposits**	
Which nodal stations should be considered as “regional” versus “non-regional”?	• Regional lymph nodes (that together define the cN stage) include mesorectal nodes and nodes in the mesocolon of the distal sigmoid colon (including nodes along the superior rectal artery and vein), obturator nodes, and internal iliac nodes. • Non-regional lymph nodes (to be considered as part of the cM stage) include external iliac and common iliac nodes. • Inguinal lymph nodes are typically considered non-regional (cM stage) nodes. In tumors extending into the anal canal below the level of the dentate line, inguinal nodes may still be considered regional / cN-stage nodes (as indicated by the AJCC-TNM8). • Radiologists should specify the location of suspicious regional lymph nodes and explicitly mention the presence of any cN+ nodes along the superior rectal artery/vein (incl. the level of the most proximal suspicious lymph node) and in the obturator and internal iliac space to inform proper radiotherapy and surgical treatment planning. • Obturator, internal iliac, and external iliac nodes are commonly referred to as the “lateral nodes.” The anatomical map in Fig. 4 can serve as a support tool to anatomically define these lateral lymph node stations on MRI.
Which criteria to use for characterization of lateral lymph nodes?	• At primary staging, a threshold of ≥ 7 mm (short-axis diameter) may be used as a criterion to diagnose cN+ nodes in the obturator and internal iliac compartments (as proposed by the Lateral Node Consortium [26]). • Unlike in mesorectal nodes, morphologic criteria (shape, border contour, signal heterogeneity) should *not* be taken into account to stage lateral lymph nodes [27]. • The panel does not support the thresholds of > 4 mm (internal iliac) and > 6 mm (obturator) to diagnose yN+ nodes post-CRT as proposed by the Lateral Node Study Consortium [26], as the evidence provided is not strong enough to warrant clinical adoption at this point. ° The panel, however, acknowledges that at the time of writing there is no alternative evidence available to suggest different criteria. Hence, clinicians may choose to take the criteria proposed by the Lateral Node Study Consortium into account. Patients with potentially suspicious lateral nodes post-CRT should always be discussed individually by a multidisciplinary team.
How to report and differentiate lymph nodes versus tumor deposits on imaging?	• There is to date insufficient evidence to know whether imaging can accurately differentiate between lymph nodes and tumor deposits. • The COMET trial (UK) is currently investigating specific criteria to discriminate between lymph nodes and tumor deposits on MRI [24]. The results of this trial should be awaited to prove if these criteria are reproducible, accurate, and prognostically significant and should thus be routinely adopted for radiological staging • Meanwhile, the panel advises to report any nodules discontinuous from the tumor (regardless whether considered as nodes or deposits) as part of the cN stage and to provide a prose description of the size and aspect of these lesions in the report.
**Definition of cM1a disease**	
How to define cM stage in case of metastases in paired organs?	• cM1a disease is defined as the presence of metastatic disease in only one site/organ. Multiple metastases within one organ, even if the organ is paired (lungs, ovaries, kidneys), still constitutes M1a disease.

## Discussion

Results of a global online survey with > 300 respondents on the application of TNM8 for the radiological staging of rectal cancer revealed several problem areas where TNM definitions are either ambiguous or difficult to apply to a radiological setting. Some problem areas were identified that mainly occurred for less experienced respondents, indicating a need for further education.

### cT staging in low-rectal cancers involving the anal canal

cT staging in tumors involving the anal canal was among the topics that reached the least agreement (45–73%) between respondents. Definitions on how to incorporate anal involvement into cT stage are either not reported or vary between different TNM editions [3, 4]. The TNM system is primarily driven by prognostic outcome stratification, and evidence on how invasion into different layers of the anal canal translates into patient outcomes is largely lacking. Although several classification systems to address low-rectal cancer have been proposed [5, 6], none have been unanimously adopted into guidelines. There is now a growing tendency among professional societies to use descriptive prose to inform clinicians about involvement of the anal canal, rather than to rely solely on cT stage. This is a strategy that was also strongly supported by our panel. The panel further agreed that cT staging should primarily be informed by the extent of tumor invasion at the level of the rectum and that involvement of the internal anal sphincter and intersphincteric space should not be taken into account in cT-stage categorization. Considering that pathologists consider skeletal muscle invasion as pT4b disease and aiming to avoid inconsistencies between radiology and pathology reports, the panel agreed that involvement extending into the external anal sphincter, puborectalis, or levator ani muscles (i.e., skeletal muscles) should be classified as cT4b. The panel also stressed the need for good-quality MRI, including a high-resolution coronal T2-weighted sequence parallel to the anal canal. Finally, the panel recommended to include a statement or suffix in the conclusion of the radiological report when there is involvement of the anal canal (e.g., “anal+”) and to provide a detailed prose description on the extent of invasion in the body of the report given the evident impact on surgical treatment [7, 8] and radiotherapy planning [9].

### Definitions of cT4b disease

The survey included a case with tumor invasion beyond the MRF into the fat of the obturator space; 57% of respondents considered this as deep cT3 infiltration, while 15% classified this as cT4b disease. This discrepancy can be explained by the fact that TNM does not provide a clear definition of what is covered by the umbrella term “structures” in their classification of cT4b disease as “any tumour with invasion of another organ or *structure*.” The panel agreed that from a surgical point of view, cT4b disease should include any tumor with direct invasion of either another organ and/or any anatomical compartment or structure (except peritoneum alone) outside the mesorectum, as this would require adaptation of the standard surgical resection plane. This rendered the proposed definitions for cT4b disease as outlined in Table 3.

### Definitions for MRF involvement

The tumor-MRF distance is sometimes referred to by radiologists as the “circumferential resection margin” (CRM), which is not accurate. Unlike MRF, which is an anatomical term, the CRM is the margin the surgeon creates when performing a resection, and what pathologists report when describing the smallest distance between the tumor and the outer plane of the resected specimen. Ideally, this plane will be along the MRF, but the CRM may be smaller when the MRF is breached during surgery or wider when the resected specimen includes additional tissue outside the MRF. In such cases, the MRF may be free of tumor but with an involved CRM, or vice versa. To avoid confusion, radiologists should therefore not use CRM but describe the tumor in relation to the MRF [10].

Respondents reached ≥ 80% agreement that macroscopic MRF invasion (i.e., a 0-mm margin) defines an involved MRF, but cases with a margin of ≤ 1 mm or 1–2 mm lacked clear consensus. In most guidelines, a cut-off of ≤ 1 mm is currently adopted as a criterion for MRF involvement [2, 11–13]. A pathology report from Nagtegaal et al (note: describing CRM and not MRF margins) proposed a cut-off of ≤ 2 mm as these tumors still show a significantly increased risk for local recurrence (16% versus 6% for tumors with a > 2-mm margin), although tumors with a ≤ 1-mm margin clearly constituted the highest-risk group (36% local recurrences) [14]. The consensus guidelines on rectal MRI published by the European Society of Gastrointestinal and Abdominal Radiology (ESGAR) proposed a margin of ≤ 1 mm to define an involved MRF but also mentioned a margin of 1–2 mm (or ≤ 2 mm) as a “threatened” MRF [15]. This sub-classification has not been widely adopted, and according to the survey results, these ambiguous definitions are a potential source of confusion. The panel therefore agreed to adopt the ≤ 1-mm threshold as a uniform criterion to define an involved MRF, and discard the definition of a threatened MRF.

A second identified problem was that there are no validated definitions on how to classify MRF involvement by tumor-bearing structures other than the primary tumor. As outlined in a review by Gollub et al [1], pathologic lymph nodes causing positive margins at histopathology do not confer an added risk for local recurrence compared to control cases with non-involved margins [14]. Moreover, it is uncommon that mesorectal lymph nodes are the only factor responsible for margin involvement on histopathological examination [16]. Conclusive data on the prognostic importance of margin involvement by tumor deposits or extramural vascular invasion (EMVI) are currently not available, although a study by Birbeck et al suggested that margin involvement caused by EMVI or tumor deposits adds a 20% and 31% risk for local recurrence, respectively, versus a 42% added risk for direct tumor invasion [17].

Current guidelines do not include any specific recommendations on whether to stratify patients for neoadjuvant treatment based on MRF involvement by the primary tumor or by nodes, deposits, or EMVI, recognizing that further studies are strongly needed. The panel agreed that for now the MRF should be considered as involved in case of a margin of ≤ 1 mm from either the primary tumor; any irregularly enlarged lymph nodes or tumor deposits; or EMVI. The panel also recommended that radiologists should no longer consider the MRF as involved when potentially malignant smooth enlarged lymph nodes (i.e., with an apparently intact capsule) are contacting the MRF. The panel considered the prognostic implications of these nodes as low and recognized the risk of overstaging and potential overtreatment in such cases, considering the limited accuracy of MRI for nodal staging [18, 19]. Finally, the panel agreed that MRF involvement should be included in the conclusion of the radiology report indicated as a suffix (or description) which specifies whether invasion is caused by the primary tumor or other structures, e.g., “MRF+ (primary)” or “MRF+ (non-primary)”.

### MRF involvement and cT staging

There was insufficient agreement (73–79%) among survey respondents for cT staging in cases with MRF versus peritoneal invasion. As outlined in Fig. 3, the mesorectum is fully covered by the MRF below the anterior peritoneal reflection. The MRF is a separate anatomical structure and not a synonym for peritoneum. MRF involvement should thus be classified as cT3 MRF+ and not cT4a disease (as erroneously done by 22% of respondents). At and above the level of the peritoneal reflection, the mesorectum is partly covered by peritoneum (anteriorly). When there is anterior invasion at these levels, this constitutes cT4a disease and the MRF should not be classified as involved (as erroneously done by 41% of respondents), except when there is simultaneous invasion of the MRF dorsally (i.e., cT4a MRF+). The suboptimal agreement in the survey results indicates a knowledge gap requiring further teaching, supported by the fact that the most experienced respondents did reach consensus in these cases.

### Lymph nodes and tumor deposits

Definitions of what constitutes a node or a deposit vary between different TNM editions [2, 20, 21], and the appropriateness of these definitions has been argued extensively. A meta-analysis of histopathology data demonstrated that, though tumor deposits correlated with the presence of lymph nodes and EMVI, they have distinctly different prognostic implications [22]. In a recent Delphi-consensus study, an international panel of pathologists agreed that tumor deposits are prognostically worse than lymph node metastases and that the N1c staging position as outlined in TNM8 is suboptimal as it does not properly reflect this risk status in the staging hierarchy [23].

Clear guidelines on how the presence of tumor deposits versus or in addition to nodal metastases should impact treatment stratification are also lacking, although in general both are considered adverse prognostic features that frequently imply a necessity for some form of (neo)adjuvant treatment. In line with the inconsistency in TNM definitions, validated definitions on what defines a lymph node or tumor deposit on imaging are lacking. The UK group of Brown et al have proposed a definition where tumor deposits are classified as “discontinuous EMVI” and characterized as nodules arising within/along venous channels, in continuity with major venous branches within the mesorectum and discontinuous from the main tumor, while nodes are characterized by the familiar shape and capsule typical of lymph nodes. The COMET trial is currently investigating the reproducibility of these definitions and the concordance between MRI and histopathology, along with the prognostic implications [24]. The panel agreed that we need to await the results of this trial to discover if the proposed criteria are reproducible and prognostically significant enough to warrant adoption into routine radiological reporting. Meanwhile, the panel proposes that any nodules discontinuous from the tumor (regardless of whether considered as nodes or deposits) are included in the cN-stage category and a prose description of the size and morphology of these lesions should be included in the report.

### Lateral lymph nodes

According to TNM definitions, any nodes within the mesorectum and in the distal sigmoid mesocolon, as well as nodes in the obturator space and alongside the internal iliac vessels, are considered regional lymph nodes. Although these nodes are all embedded in the N stage, the panel unanimously agreed that further specification of which regional lymph node stations are involved is important to inform surgical and radiotherapy planning. Specifically, the presence of “high” lymph nodes along the superior rectal blood vessels impacts the upper borders of the radiotherapy volume [9] while N+ nodes in the “lateral” (obturator, internal iliac) compartments are associated with a higher risk for local recurrence, which can be improved by lateral lymph node dissection and/or targeted (chemo)radiotherapy [25]. As such, these nodes should be mentioned explicitly. Lymph nodes along the external iliac vessels are also considered part of the lateral nodes, but like lymph nodes along the common iliac vessels, lymph node involvement is much less common in these regions and would constitute non-regional (M1-stage) nodal disease. Elongated (oval) nodes in the posterior external iliac compartment, i.e., directly dorsal to the external iliac vein, are commonly encountered on MRI and have been demonstrated to be of little or no clinical significance [1]. Inguinal lymph nodes are typically also considered non-regional nodes. As an exception, the AJCC version of the TNM specifies that for distal tumors extending below the dentate line, inguinal nodes should be considered regional nodes similar to anal cancer staging.

Despite these relatively straightforward definitions, differentiation of regional versus non-regional lymph nodes was identified as an area of much disagreement in the survey results, probably reflecting a knowledge gap. A contributing factor may be the limited availability of radiological guidelines to define the various anatomical compartments for nodal staging on MRI. In an online supplement to a publication in JAMA surgery, Ogura et al published a color map defining the lateral lymph node compartments on MRI [26]. In Fig. 4, the panel proposed a slightly adapted version of this map with specified oblique-axial views (as typically encountered during radiological staging), also considering previously published definitions from the radiation oncologists society [9]. The panel believes that such maps can offer useful support to radiologists and can contribute to improved consistency in reporting of lateral lymph nodes.

Evidence on which criteria to use for evaluation of lateral lymph nodes is very limited. In the most recent consensus publication from ESGAR, the panel proposed specific criteria based on a combination of size and morphology features for mesorectal nodes, but acknowledged that for lateral lymph nodes, no specific criteria could be derived from literature at that time [15]. Subsequently, the Lateral Node Study Consortium published a pooled retrospective multicenter analysis of 741 patients, proposing a cut-off of ≥ 7 mm for obturator and internal iliac nodes at primary staging to define cN+ nodes, combined with a cut-off of > 4 mm (internal iliac) and > 6 mm (obturator) after chemoradiotherapy as criteria associated with a higher-than-acceptable risk for lateral lymph node recurrence [26]. The same group also showed that in contrast to mesorectal nodes, morphologic features are not of added benefit for lateral nodal staging [27]. Considering the current level of evidence, the panel agreed that for primary staging, the ≥ 7-mm threshold may for now be adopted, although further validation is obviously needed. The panel did not support the proposed size thresholds after chemoradiotherapy as the evidence provided was considered too preliminary. Reasons for concern included under-investigation of confounding effects (e.g., varying intervals between neoadjuvant treatment and radiological re-assessment/surgery, varying radiation volumes/doses). Nevertheless, the panel acknowledged that at the moment no alternative criteria are available.

### Other (non-TNM) staging controversies

The authors acknowledge that there are several other potential controversies in the radiological staging of rectal cancer that are not (or less directly) related to the TNM-staging system and were therefore outside the scope of the current paper. These include the radiological classification of mucinous tumors, MRI protocols and patient preparation, criteria for restaging after neoadjuvant treatment, and the anatomical localization of tumors (including the differentiation between distal-, mid-, and high-rectal cancer, and the classification of tumors near the rectosigmoid junction as either rectal or sigmoid). With respect to the latter, the authors would like to refer to recent publications describing the “sigmoid take-off” as a useful radiological landmark (recently agreed upon by expert consensus) to discriminate rectal from sigmoid cancer [28, 29]. Regarding the differentiation between distal-, mid-, and high-rectal cancer, it is mainly the management of high-rectal cancers that in some countries (like the USA) is different and usually does not involve neoadjuvant treatment. Though there are no unanimously agreed upon definitions, the anterior peritoneal reflection is a commonly used anatomical landmark that can also easily be recognized on MRI [30].

In conclusion, this paper provides recommendations derived from the outcome of a global online survey and discussed among a panel of recognized multidisciplinary experts in the field on how to handle current controversies in TNM-based staging of rectal cancer on MRI related to cT staging in low-rectal cancers, definitions for cT4b disease and MRF invasion, evaluation of tumor deposits versus nodes, and the staging of lateral lymph nodes. These recommendations may serve as a practice guide and support tool for radiologists (and other clinicians) involved in the staging of rectal cancer, help guide multidisciplinary team discussions, and will hopefully contribute to improved consistency in radiological reporting.

## Supplementary Information

ESM 1 (DOCX 1.95 MB)

## Abbreviations

cN stage: Clinical nodal stage
CRM: Circumferential resection margin
CT: Computed tomography
cT stage: Clinical tumor stage
EMVI: Extramural vascular invasion
ESGAR: European Society of Gastrointestinal and Abdominal Radiology
MRF: Mesorectal fascia
MRI: Magnetic resonance imaging
TNM: Tumor node metastases

## Declarations

### Guarantor

The scientific guarantor of this publication is Doenja M.J. Lambregts.

### Statistics and Biometry

No complex statistical methods were necessary for this paper.

### Methodology

• prospective

• Survey and consensus study

• Multicenter study

## References

[1] Gollub MJ, Lall C, Lalwani N, Rosenthal MH (2019). Current controversy, confusion, and imprecision in the use and interpretation of rectal MRI. Abdom Radiol (NY).

[2] Jessup MJ, Goldberg RM, Asare EA, et al (2017) Colon and rectum. In: Amin MB, Edge S, Greene F, Byrd DR, Brookland RK, Washington MK, et al Eds. AJCC cancer staging manual (8th edition). Springer: 251–273

[3] Wittekind C, Greene FL, Henson DE, et al (2003) Explanatory notes specific anatomical sites. In: Wittekind Ch, Greene F.L, Henson D.E et al eds. TNM supplement: a commentary on uniform use 3^rd^ edition. New York, NY. Wiley-Liss: 40–86

[4] Wittekind C, Brierly JD, Lee AWM, et al (2019) Explanatory notes specific anatomical sites. In: Wittekind C, Brierly J.D, Lee A.W.M, et al eds. TNM supplement: a commentary on uniform use. 5^th^ edition. New York, NY. Wiley-Liss: 54–85

[5] Shihab OC, How P, West N (2011). Can a novel MRI staging system for low rectal cancer aid surgical planning?. Dis Colon Rectum.

[6] Bamba Y, Itabashi M, Kameoka S (2012). Preoperative evaluation of the depth of anal canal invasion in very low rectal cancer by magnetic resonance imaging and surgical indications for intersphincteric resection. Surg Today.

[7] You YN, Hardiman KM, Bafford A (2020). The American Society of Colon and Rectal Surgeons Clinical Practice Guidelines for the Management of Rectal Cancer. Dis Colon Rectum.

[8] Bleday R, Melnitchouk N (2014) Surgical management of rectal cancer. In: Beck, D.E., Nasseri, Y., Hull, et al eds. The ASCRS manual of colon and rectal surgery (2^nd^ ed.). Springer: 811–831

[9] Valentini V, Gambacorta MA, Barbaro B (2016). International consensus guidelines on clinical target volume delineation in rectal cancer. Radiother Oncol.

[10] Glimelius B, Beets-Tan R, Blomqvist L (2011). Mesorectal fascia instead of circumferential resection margin in preoperative staging of rectal cancer. J Clin Oncol.

[11] Glynne-Jones R, Wyrwicz L, Tiret E, et al (2017) Rectal cancer: ESMO clinical practice guidelines for diagnosis, treatment and follow-up. Ann Oncol 28(suppl_4):iv22–iv40 10.1093/annonc/mdx224 28881920

[12] National Comprehensive Cancer Network. Rectal cancer (version 1.2021) [www.nccn.org] Available at: https://www.nccn.org/professionals/physician_gls/pdf/rectal.pdf. Accessed June 21, 2021

[13] Federatie Medische specialisten. Richtlijn colorectaal carcinoom (2019 update). Available at: https://richtlijnendatabase.nl/richtlijn/colorectaal_carcinoom_crc/startpagina_-_crc.html. Accessed December 2, 2020

[14] Nagtegaal ID, Marijnen CA, Kranenbarg EK, van de Velde CJ, van Krieken JH, Pathology Review Committee; Cooperative Clinical Investigators (2002). Circumferential margin involvement is still an important predictor of local recurrence in rectal carcinoma: not one millimeter but two millimeters is the limit. Am J Surg Pathol.

[15] Beets-Tan RGH, Lambregts DMJ, Maas M (2019). Magnetic resonance imaging for clinical management of rectal cancer: updated recommendations from the 2016 European Society of Gastrointestinal and Abdominal Radiology (ESGAR) consensus meeting. Eur Radiol.

[16] Shihab OC, Quirke P, Heald RJ, Moran BJ, Brown G (2010). Magnetic resonance imaging-detected lymph nodes close to the mesorectal fascia are rarely a cause of margin involvement after total mesorectal excision. Br J Surg.

[17] Birbeck KF, Macklin CP, Tiffin NJ (2002). Rates of circumferential resection margin involvement vary between surgeons and predict outcomes in rectal cancer surgery. Ann Surg.

[18] Bipat S, Glas AS, Slors FJ (2004). Rectal cancer: local staging and assessment of lymph node involvement with endoluminal US, CT, and MR imaging—a meta-analysis. Radiology.

[19] Lahaye MJ, Engelen SM, Nelemans PJ (2005). Imaging for -predicting the risk factors—the circumferential resection margin and nodal disease—of local recurrence in rectal cancer: a meta-analysis. Semin Ultrasound CT MR.

[20] Fleming ID, Cooper JS, Henson DE, et al (1997) Eds. General information on cancer staging and end-results reporting. In: AJCC Cancer Staging Manual (5^th^ edition). Lippincott-Raven: 3–11

[21] Greene FL, Page DL, Fleming ID, et al (2002). Eds. AJCC cancer staging manual (6^th^ edition). Springer

[22] Nagtegaal ID, Knijn N, Hugen N (2017). Tumour deposits in colorectal cancer: improving the value of modern staging-a systematic review and meta-analysis. J Clin Oncol.

[23] Lord A, Brown G, Abulafi M (2021). Histopathological diagnosis of tumour deposits in colorectal cancer: a Delphi consensus study. Histopathology.

[24] Lord AC, Moran B, Abulafi M (2020). Can extranodal tumour deposits be diagnosed on MRI? Protocol for a multicentre clinical trial (the COMET trial). BMJ Open.

[25] Schaap DP, Boogerd LSF, Konishi T (2021). Lateral node study consortium. Rectal cancer lateral lymph nodes: multicentre study of the impact of obturator and internal iliac nodes on oncological outcomes. Br J Surg.

[26] Ogura A, Konishi T, Cunningham C et al (2019) Lateral nodal features on restaging magnetic resonance imaging associated with lateral local recurrence in low rectal cancer after neoadjuvant chemoradiotherapy or radiotherapy. JAMA Surg 154(9):e192172. 10.1001/jamasurg.2019.2172 10.1001/jamasurg.2019.2172 PMC6613303 31268504

[27] Ogura A, Konishi T, Cunningham C, et al Lateral Node Study Consortium (2019) Neoadjuvant (chemo)radiotherapy with total mesorectal excision only is not sufficient to prevent lateral local recurrence in enlarged nodes: results of the multicenter lateral node study of patients with low cT3/4 rectal cancer. J Clin Oncol 37(1):33–43 10.1200/JCO.18.00032 PMC6366816 30403572

[28] D’Souza N, de Neree Tot Babberich MPM, d’Hoore A (2019). Definition of the rectum: an international, expert-based delphi consensus. Ann Surg.

[29] Bogveradze N, Lambregts DMJ, El Khababi N et al (2021) The sigmoid take-off as a landmark to distinguish rectal from sigmoid tumours on MRI: reproducibility, pitfalls and potential impact on treatment stratification. Eur J Surg Oncol 20:S0748-7983(21)00735–6. 10.1016/j.ejso.2021.09.009 10.1016/j.ejso.2021.09.009 34583878

[30] Gollub MJ, Maas M, Weiser M (2013). Recognition of the anterior peritoneal reflection at rectal MRI. AJR Am J Roentgenol.

