# Supplementary material for: Current controversies in TNM for the radiological staging of rectal cancer and how to deal with them: results of a global online survey and multidisciplinary expert consensus
Source: Eur Radiol. 2022 Mar 7;32(7):4991–5003. doi: 10.1007/s00330-022-08591-z (PMC9213337; doi:10.1007/s00330-022-08591-z)
Supplement: Supplementary file 1 — (DOCX 1.95 MB) [file 330_2022_8591_MOESM1_ESM.docx]

**Supplement 1** Full case-based survey

**Section 1 – T-staging**

**Case 01: below you see a tumour limited to the bowel wall. What is the cT-stage:**
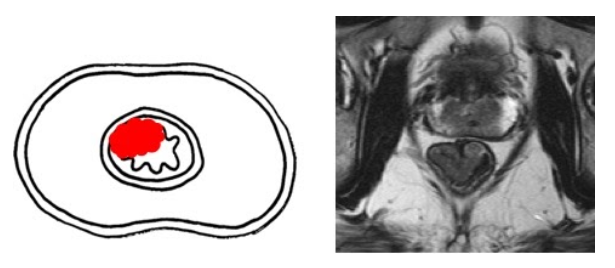


Answer options:

○ cT1 ○ cT3

○ cT2 ○ cT4a

○ cT1-2 (unable to differentiate between cT1 and cT2) ○ cT4b

○ I do not know

**Case 02: Below you see a tumour that extends beyond the bowel wall and grows into the perirectal fat. The mesorectal fascia is intact. What is the cT-stage:**


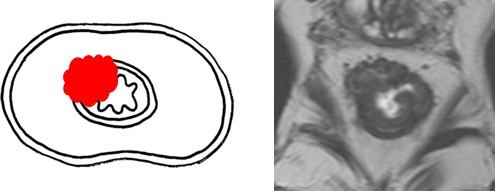


Answer options:

○ cT1 ○ cT3

○ cT2 ○ cT4a

○ cT1-2 (unable to differentiate between cT1 and cT2) ○ cT4b

○ I do not know

**Case 03: Below you see a tumour that invades the seminal vesicles and part of peripheral zone of the prostate. What is the cT-stage:**


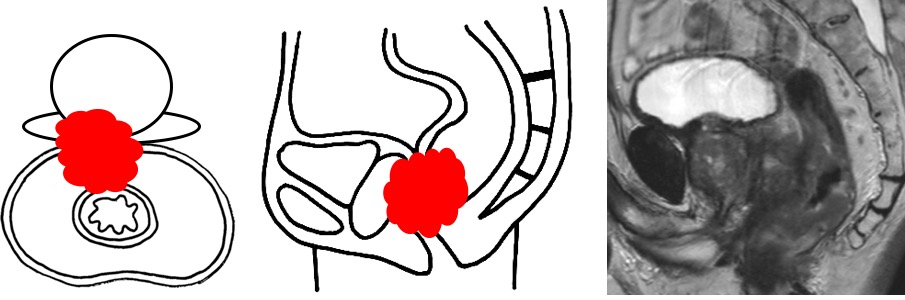


Answer options:

○ cT1 ○ cT3

○ cT2 ○ cT4a

○ cT1-2 (unable to differentiate between cT1 and cT2) ○ cT4b

○ I do not know

**Case 04: Below you see a tumour that grows beyond the rectal wall and invades the mesorectal fascia. It does not invade any other organs or structures. What is the cT-stage:**

**
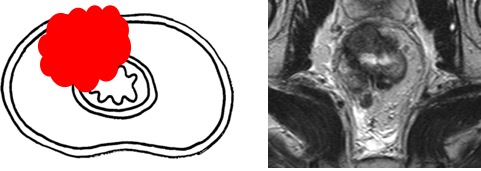
**

Answer options:

○ cT1 ○ cT3

○ cT2 ○ cT4a

○ cT1-2 (unable to differentiate between cT1 and cT2) ○ cT4b

○ I do not know

**Case 05: Below you see a tumour that invades the anterior peritoneal reflection. What is the cT-stage:**

**
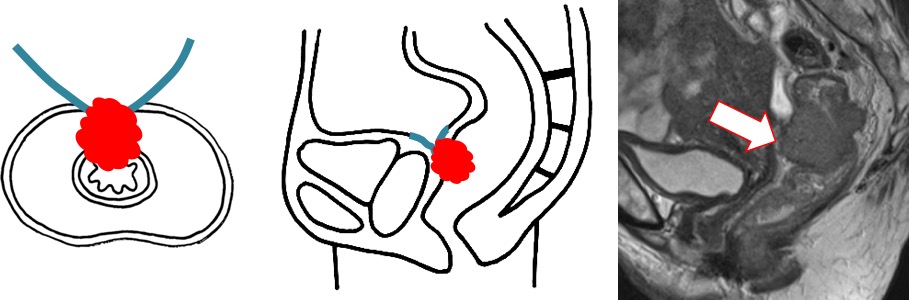
**

Answer options:

○ cT1 ○ cT3

○ cT2 ○ cT4a

○ cT1-2 (unable to differentiate between cT1 and cT2) ○ cT4b

○ I do not know

**Case 06: Below you see a tumour that invades the peritoneum above the level of the anterior peritoneal reflection. What is the cT-stage:**

**
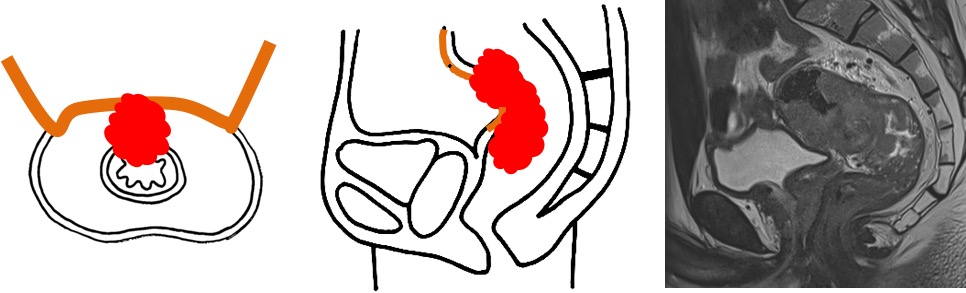
**

Answer options:

○ cT1 ○ cT3

○ cT2 ○ cT4a

○ cT1-2 (unable to differentiate between cT1 and cT2) ○ cT4b

○ I do not know

**Case 07: Below you see a tumour that grows beyond the mesorectal fascia into the fat of the obturator space (where it does not invade any muscles or vessels). What is the cT-stage:**

**
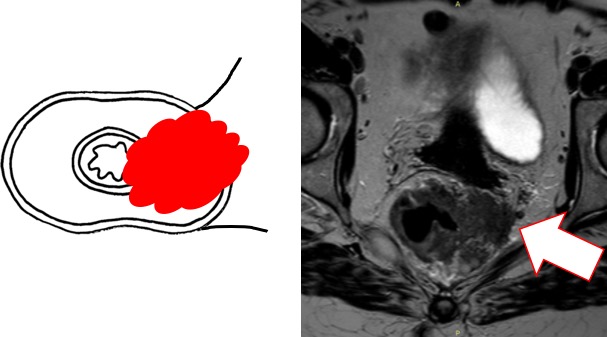
**

Answer options:

○ cT1 ○ cT3

○ cT2 ○ cT4a

○ cT1-2 (unable to differentiate between cT1 and cT2) ○ cT4b

○ I do not know

**Section 2 – Anal sphincter and pelvic floor invasion**

**Case 08: Below you see a rectal tumour that extends into the anal canal where it grows into the internal anal sphincter. The intersphincteric plane and external sphincter are not involved. What is the cT-stage:**

**
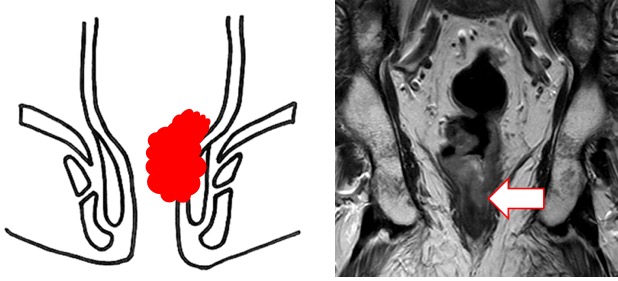
**

Answer options:

○ cT1 ○ cT3

○ cT2 ○ cT4a

○ cT1-2 (unable to differentiate between cT1 and cT2) ○ cT4b

○ I do not know

**Case 09: Below you see a rectal tumour that extends into the anal canal where it grows through the internal sphincter and invades the intersphincteric plane. The external anal sphincter is not involved. What is the cT-stage:**

**
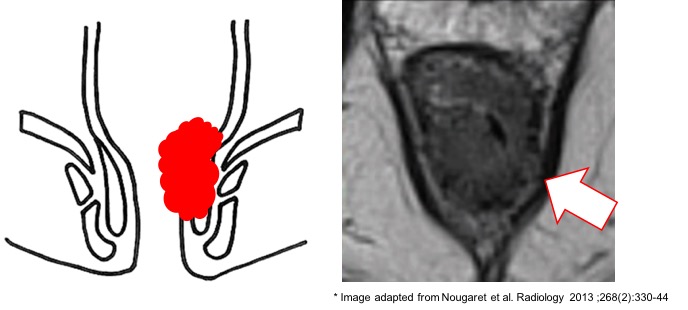
**

Answer options:

○ cT1 ○ cT3

○ cT2 ○ cT4a

○ cT1-2 (unable to differentiate between cT1 and cT2) ○ cT4b

○ I do not know

**Case 10: Below you see a rectal tumour that extends into the anal canal where it grows beyond the internal sphincter and intersphincteric plane and invades the external sphincter. What is the cT-stage:**

**
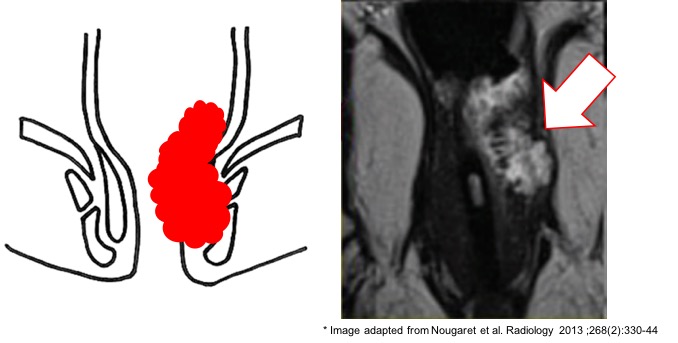
**

Answer options:

○ cT1 ○ cT3

○ cT2 ○ cT4a

○ cT1-2 (unable to differentiate between cT1 and cT2) ○ cT4b

○ I do not know

**Case 11: Below you see a rectal tumour that extends beyond the rectal wall at the level of the rectum (above the level of the anorectal junction) and grows into the levator ani muscle (pelvic floor). What is the cT-stage:**


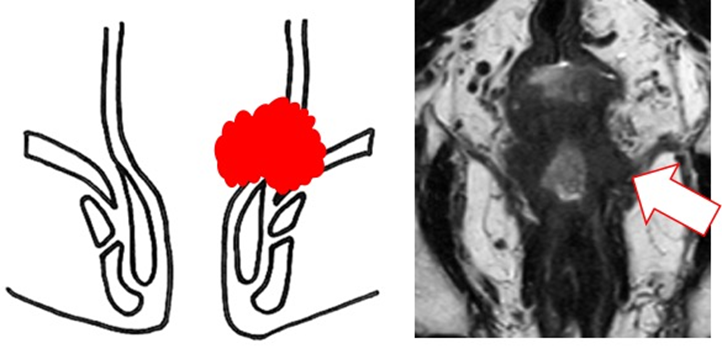


Answer options:

○ cT1 ○ cT3

○ cT2 ○ cT4a

○ cT1-2 (unable to differentiate between cT1 and cT2) ○ cT4b

○ I do not know

**Section 3 – Mesorectal Fascia (MRF) involvement**

**Case 12: Below you see a distal rectal tumour that extends beyond the rectal wall BELOW the level of the anterior peritoneal reflection. The distance between the tumour and MRF is 0 mm. What is the MRF status:**


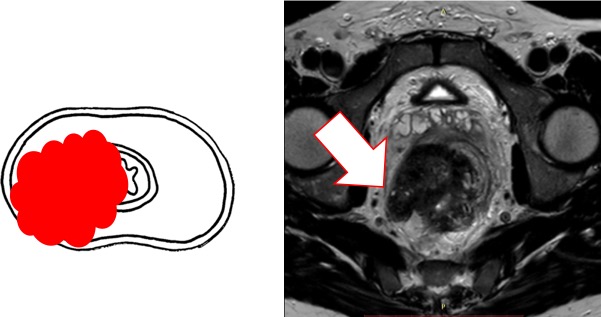


Answer options:

○ MRF is involved

○ MRF is not involved

○ Doubtful / I do not know

**Case 13: Below you see a distal rectal tumour that extends beyond the rectal wall BELOW the level of the anterior peritoneal reflection. The distance between tumour and MRF is < 1 mm. What is the MRF status:**


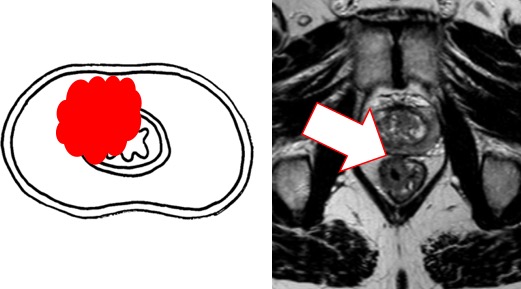


Answer options:

○ MRF is involved

○ MRF is not involved

○ Doubtful / I do not know

**Case 14: Below you see a distal rectal tumour that extends beyond the rectal wall BELOW the level of the anterior peritoneal reflection. The distance between tumour and MRF is 1-2 mm. What is the MRF status:**


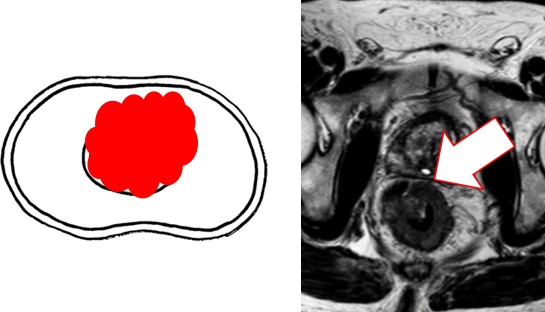


Answer options:

○ MRF is involved

○ MRF is not involved

○ Doubtful / I do not know

**Case 15: Below you see a proximal rectal tumour that extends beyond the rectal wall ABOVE the level of the peritoneal reflection. ANTERIORLY it invades the peritoneum. What is the MRF status:**


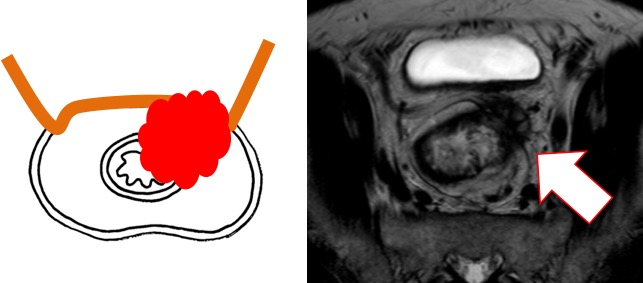


Answer options:

○ MRF is involved

○ MRF is not involved

○ Doubtful / I do not know

**Case 16: Below you see a proximal rectal tumour that extends beyond the rectal wall POSTERIORLY, ABOVE the level of the anterior peritoneal reflection (margin to MRF 0 mm). What is the MRF status:**


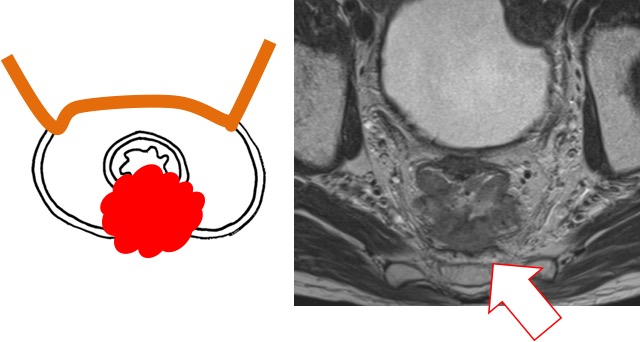


Answer options:

○ MRF is involved

○ MRF is not involved

○ Doubtful / I do not know

**Case 17: Below you see a suspicious lymph node directly adjacent to the MRF. The node is sharply delineated without any signs of extracapsular extension. What is the MRF status:**


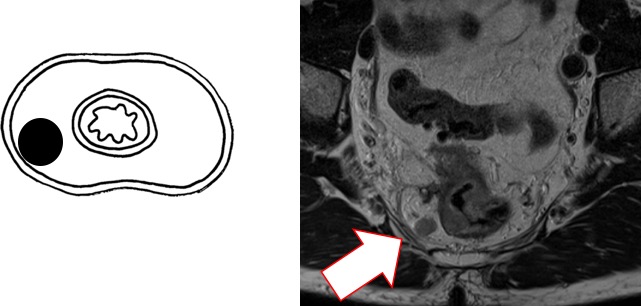


Answer options:

○ MRF is involved

○ MRF is not involved

○ Doubtful / I do not know

**Case 18: Below you see an irregular node (with extracapsular extension) directly adjacent to the MRF. What is the MRF status:**

**
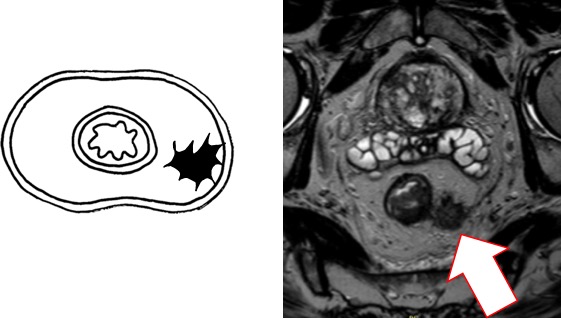
**

Answer options:

○ MRF is involved

○ MRF is not involved

○ Doubtful / I do not know

**Section 4 – Nodal staging**

**Case 19: Would you consider this to be a ...**

**
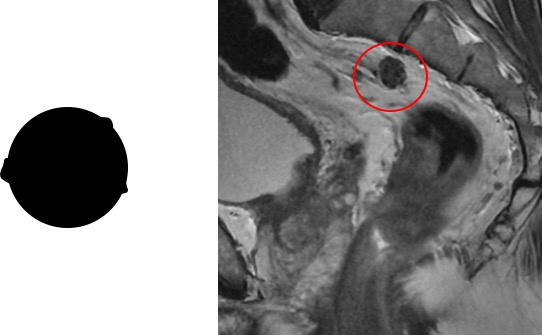
**

Answer options:

○ Pathologic lymph node

○ Tumour deposit

○ Doubtful / I do not know

**Case 20: Would you consider this to be a ...**

**
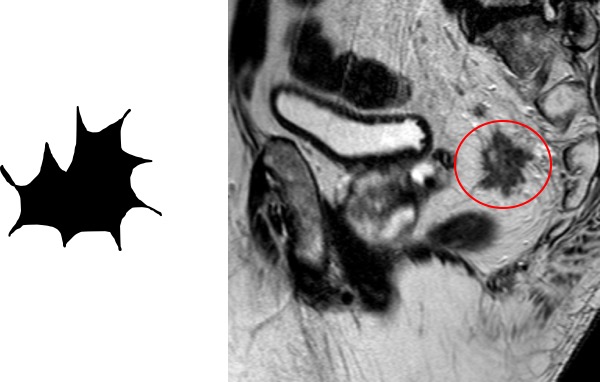
**

Answer options:

○ Pathologic lymph node

○ Tumour deposit

○ Doubtful / I do not know

**Case 21: Would you consider this to be a ...**

**
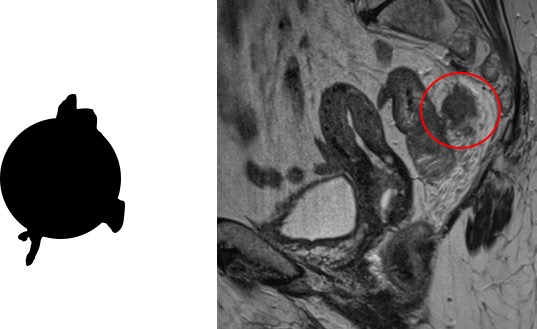
**

Answer options:

○ Pathologic lymph node

○ Tumour deposit

○ Doubtful / I do not know

**Case 22: Below you see a rectal cancer case with a single metastatic mesorectal lymph node. What is the cN-stage:**


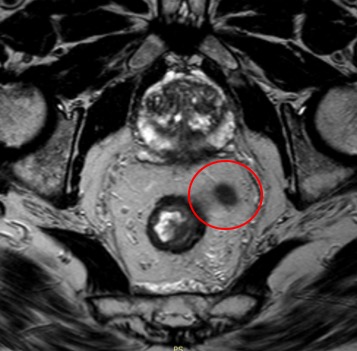


Answer options:

○ cN1a ○ cN2a

○ cN1b ○ cN2b

○ cN1c ○ I do not know

**Case 23: Below you see a rectal cancer case with two metastatic mesorectal lymph nodes. What is the cN-stage:**


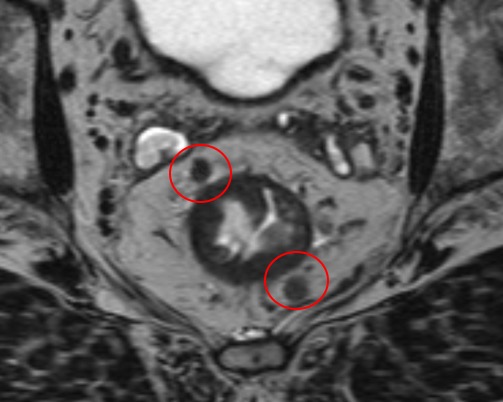


Answer options:

○ cN1a ○ cN2a

○ cN1b ○ cN2b

○ cN1c ○ I do not know

**Case 24: Below you see a rectal cancer case with a single tumour deposit in the mesorectum (there are no additional metastatic lymph nodes). What is the cN-stage:**

**
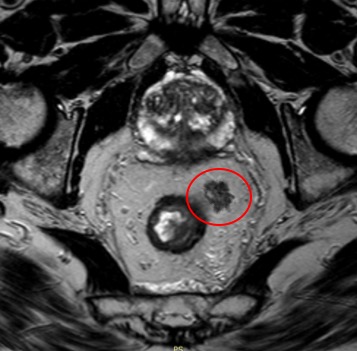
**

Answer options:

○ cN1a ○ cN2a

○ cN1b ○ cN2b

○ cN1c ○ I do not know

**Case 25: Below you a rectal cancer case with a single tumour deposit and a single metastatic lymph node in the mesorectum. What is the cN-stage:**

**
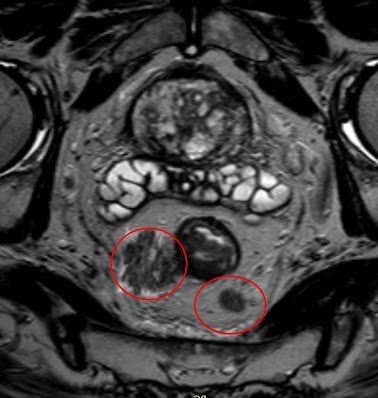
**

Answer options:

○ cN1a ○ cN2a

○ cN1b ○ cN2b

○ cN1c ○ I do not know

**Case 26: Below you see a rectal cancer case with seven metastatic mesorectal lymph nodes. What is the cN-stage:**


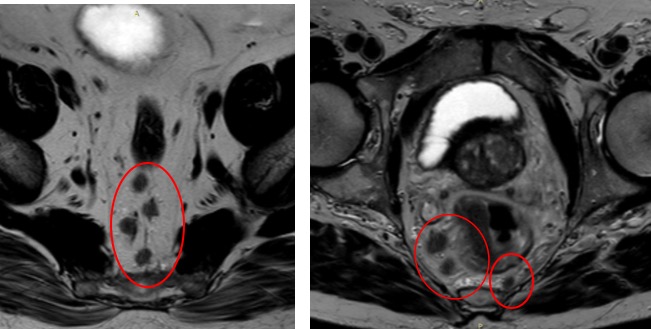


Answer options:

○ cN1a ○ cN2a

○ cN1b ○ cN2b

○ cN1c ○ I do not know

**Case 27: Below you see a rectal cancer case with four metastatic mesorectal lymph nodes. What is the cN-stage:**


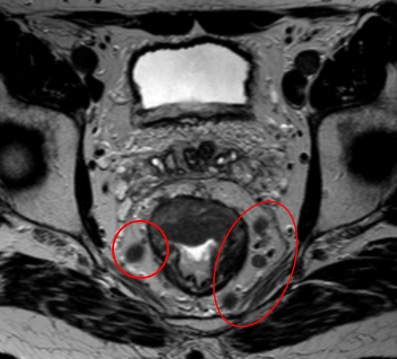


Answer options:

○ cN1a ○ cN2a

○ cN1b ○ cN2b

○ cN1c ○ I do not know

**Section 5 – Regional versus non-regional lymph nodes**

**Case 28: Below you see a rectal cancer case with a single metastatic mesorectal lymph node. What is the stage based on this node:**

**
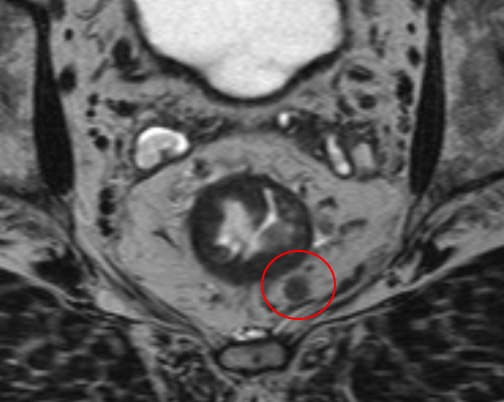
**

Answer options:

○ N1a (regional lymph node metastasis)

○ M1a (non-regional lymph node metastases)

○ I do not know

**Case 29: Below you see a rectal cancer case with a single metastatic lymph node in the obturator space. What is the stage based on this node:**

**
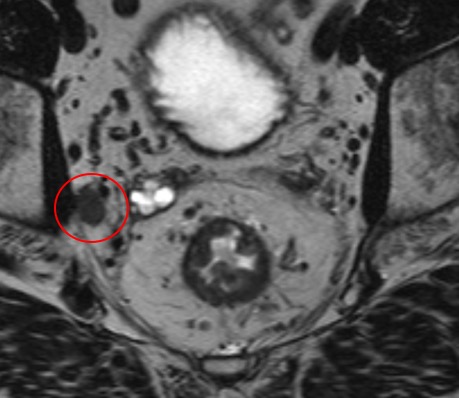
**

Answer options:

○ N1a (regional lymph node metastasis)

○ M1a (non-regional lymph node metastases)

○ I do not know

**Case 30: Below you see a rectal cancer case with a single metastatic external iliac lymph node. What is the stage based on this node:**


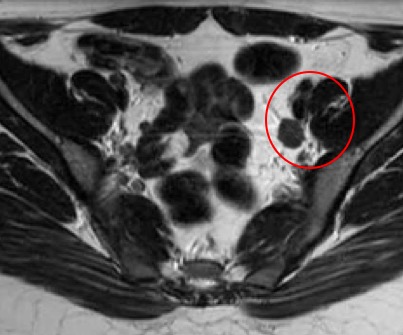


Answer options:

○ N1a (regional lymph node metastasis)

○ M1a (non-regional lymph node metastases)

○ I do not know

**Case 31: Below you see a rectal cancer case with a single metastatic internal iliac lymph node. What is the stage based on this node:**


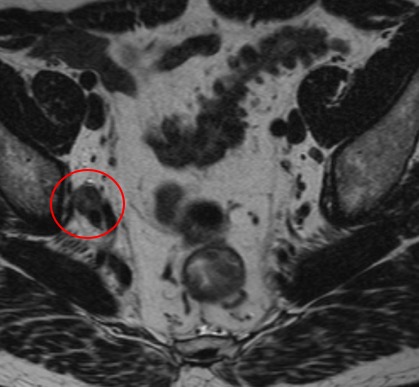


Answer options:

○ N1a (regional lymph node metastasis)

○ M1a (non-regional lymph node metastases)

○ I do not know

**Case 32: Below you see a rectal cancer case with a single metastatic common iliac lymph node. What is the stage based on this node:**


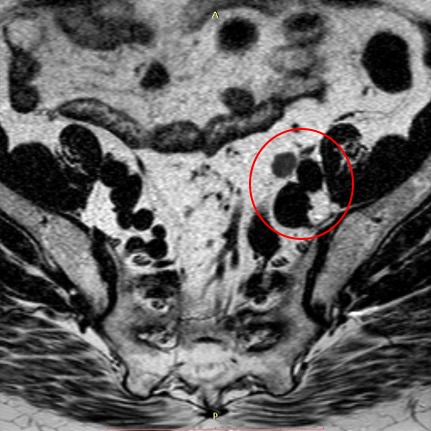


Answer options:

○ N1a (regional lymph node metastasis)

○ M1a (non-regional lymph node metastases)

○ I do not know

**Case 33: Below you see a rectal cancer case with a single metastatic superficial inguinal lymph node. The rectal tumour itself is a DISTAL TUMOUR that extends into the anal canal beyond the level of the dentate line. What is the stage based on this node:**


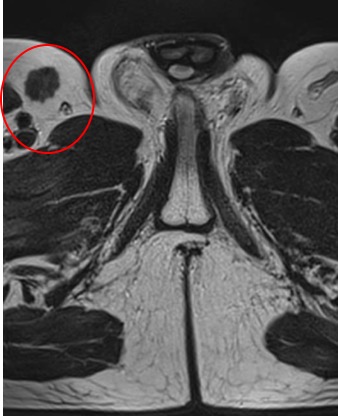


Answer options:

○ N1a (regional lymph node metastasis)

○ M1a (non-regional lymph node metastases)

○ I do not know

**Case 34: Below you see a rectal cancer case with a single metastatic superficial inguinal lymph node. The rectal tumour itself is a MID-RECTAL TUMOUR that does not extend into the anal canal. What is the stage based on this node:**


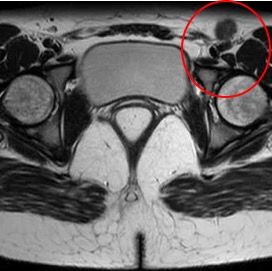


Answer options:

○ N1a (regional lymph node metastasis)

○ M1a (non-regional lymph node metastases)

○ I do not know

**Section 6 – M-staging**

**Case 35: Below you see a metastatic common iliac lymph node. What is the M-stage:**

**
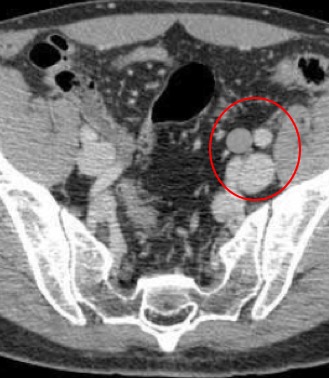
**

Answer options:

○ M1a ○ M1c

○ M1b ○ I do not know

**Case 36: Below you see liver metastases and a metastatic paraaortic lymph node. What is the M-stage:**


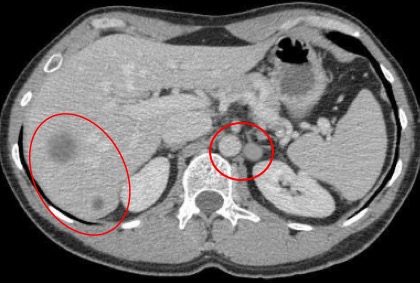


Answer options:

○ M1a ○ M1c

○ M1b ○ I do not know

**Case 37: Below you see lung metastasis that are exclusively situated in the right lung. What is the M-stage:**

**
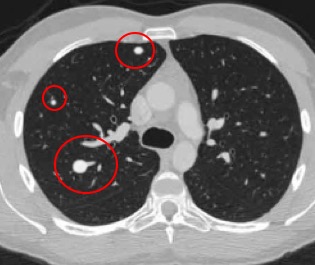
**

Answer options:

○ M1a ○ M1c

○ M1b ○ I do not know

**Case 38: Below you see a case with bilateral lung metastases. What is the M-stage:**


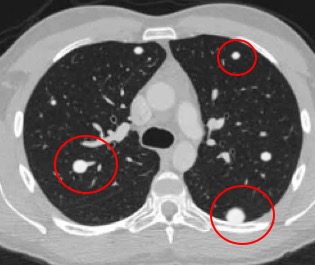


Answer options:

○ M1a ○ M1c

○ M1b ○ I do not know

**Case 39: Below you see metastases in liver, kidneys and spleen. What is the M-stage:**


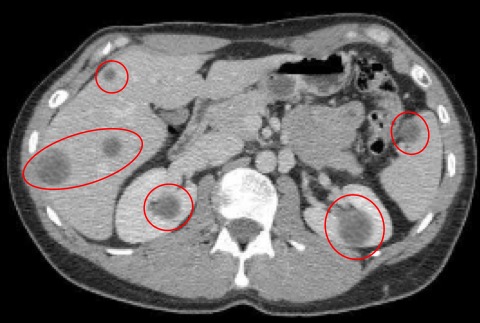


Answer options:

○ M1a ○ M1c

○ M1b ○ I do not know

**Case 40: Below you see peritoneal metastases. What is the M-stage:**


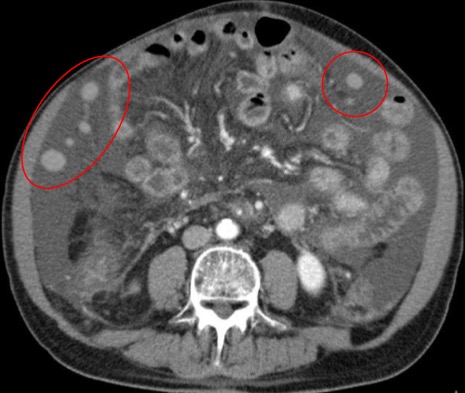


Answer options:

○ M1a ○ M1c

○ M1b ○ I do not know

**Case 41: Below you see liver and peritoneal metastases. What is the M-stage:**


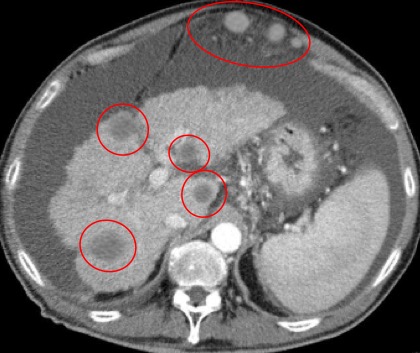


Answer options:

○ M1a ○ M1c

○ M1b ○ I do not know

| **Supplement 2** Survey results specified per profession and experience level | | | | |
| --- | --- | --- | --- | --- |
| **Section 1 – cT-staging***  Respondents were asked to assign cT-stage for each case | | | **% consensus** | |
|  | **Case 01: Tumour limited to the bowel wall (i.e., cT1-2)** | | | |
|  |  | All respondents (n=321) | 100% | cT1-2 |
|  |  | Radiologists (n=255) | 100% | cT1-2 |
|  |  | - Abdominal radiologists with specific expertise in rectal MRI (n=103) | 100% | cT1-2 |
|  |  | - Abdominal radiologists (n=87) | 100% | cT1-2 |
|  |  | - General radiologists (n=39) | 100% | cT1-2 |
|  |  | - Senior residents (n=18) | 100% | cT1-2 |
|  |  | - Junior residents (n=8) | 100% | cT1-2 |
|  |  | Non-radiologists (n=66) | 99% | cT1-2 |
|  |  | - Surgeons (n=34) | 97% | cT1-2 |
|  |  | - Radiation oncologists (n=16) | 100% | cT1-2 |
|  |  | - Pathologists (n=6) | 100% | cT1-2 |
|  |  | - Other (n=10) | 100% | cT1-2 |
|  | **Case 02: Tumour penetrating the wall and extending into perirectal fat, wide margin between tumour and MRF (i.e., cT3)** | | | |
|  |  | All respondents (n=321) | 98% | cT3 |
|  |  | Radiologists (n=255) | 100% | cT3 |
|  |  | - Abdominal radiologists with specific expertise in rectal MRI (n=103) | 100% | cT3 |
|  |  | - Abdominal radiologists (n=87) | 100% | cT3 |
|  |  | - General radiologists (n=39) | 97% | cT3 |
|  |  | - Senior residents (n=18) | 100% | cT3 |
|  |  | - Junior residents (n=8) | 100% | cT3 |
|  |  | Non-radiologists (n=66) | 92% | cT3 |
|  |  | - Surgeons (n=34) | 91% | cT3 |
|  |  | - Radiation oncologists (n=16) | 94% | cT3 |
|  |  | - Pathologists (n=6) | 100% | cT3 |
|  |  | - Other (n=10) | 90% | cT3 |
|  | **Case 03: Tumour invading the seminal vesicles and prostate (i.e., cT4b)** | |  |  |
|  |  | All respondents (n=319) | 97% | cT4b |
|  |  | Radiologists (n=254) | 98% | cT4b |
|  |  | - Abdominal radiologists with specific expertise in rectal MRI (n=103) | 100% | cT4b |
|  |  | - Abdominal radiologists (n=86) | 98% | cT4b |
|  |  | - General radiologists (n=39) | 92% | cT4b |
|  |  | - Senior residents (n=18) | 100% | cT4b |
|  |  | - Junior residents (n=8) | 100% | cT4b |
|  |  | Non-radiologists (n=65) | 97% | cT4b |
|  |  | - Surgeons (n=33) | 97% | cT4b |
|  |  | - Radiation oncologists (n=16) | 100% | cT4b |
|  |  | - Pathologists (n=6) | 83% | cT4b |
|  |  | - Other (n=10) | 100% | cT4b |
|  | **Case 04: Tumour extending into the perirectal fat, invading the MRF (i.e., cT3)** | | | |
|  |  | All respondents (n=321) | 75% | cT3 |
|  |  | Radiologists (n=255) | 79% | cT3 |
|  |  | - Abdominal radiologists with specific expertise in rectal MRI (n=103) | 86% | cT3 |
|  |  | - Abdominal radiologists (n=87) | 74% | cT3 |
|  |  | - General radiologists (n=39) | 72% | cT3 |
|  |  | - Senior residents (n=18) | 83% | cT3 |
|  |  | - Junior residents (n=8) | 63% | cT3 |
|  |  | Non-radiologists (n=66) | 58% | cT3 |
|  |  | - Surgeons (n=34) | 56% | cT3 |
|  |  | - Radiation oncologists (n=16) | 69% | cT3 |
|  |  | - Pathologists (n=6) | 67% | cT3 |
|  |  | - Other (n=10) | 50% | cT4a |
|  | **Case 05: Tumour extending into the perirectal fat, invading the anterior peritoneal reflection (i.e., cT4a)** | | | |
|  |  | All respondents (n=321) | 94% | cT4a |
|  |  | Radiologists (n=255) | 95% | cT4a |
|  |  | - Abdominal radiologists with specific expertise in rectal MRI (n=103) | 95% | cT4a |
|  |  | - Abdominal radiologists (n=87) | 95% | cT4a |
|  |  | - General radiologists (n=39) | 90% | cT4a |
|  |  | - Senior residents (n=18) | 100% | cT4a |
|  |  | - Junior residents (n=8) | 100% | cT4a |
|  |  | Non-radiologists (n=66) | 88% | cT4a |
|  |  | - Surgeons (n=34) | 88% | cT4a |
|  |  | - Radiation oncologists (n=16) | 94% | cT4a |
|  |  | - Pathologists (n=6) | 83% | cT4a |
|  |  | - Other (n=10) | 80% | cT4a |
|  | **Case 06: Tumour extending into the perirectal fat, invading the peritoneum above the peritoneal reflection (i.e., cT4a)** | | | |
|  |  | All respondents (n=321) | 89% | cT4a |
|  |  | Radiologists (n=255) | 91% | cT4a |
|  |  | - Abdominal radiologists with specific expertise in rectal MRI (n=103) | 93% | cT4a |
|  |  | - Abdominal radiologists (n=87) | 90% | cT4a |
|  |  | - General radiologists (n=39) | 87% | cT4a |
|  |  | - Senior residents (n=18) | 83% | cT4a |
|  |  | - Junior residents (n=8) | 100% | cT4a |
|  |  | Non-radiologists (n=66) | 83% | cT4a |
|  |  | - Surgeons (n=34) | 88% | cT4a |
|  |  | - Radiation oncologists (n=16) | 81% | cT4a |
|  |  | - Pathologists (n=6) | 83% | cT4a |
|  |  | - Other (n=10) | 70% | cT4a |
|  | **Case 07: Tumour extending beyond the MRF into the obturator space (without vessel or muscle invasion)** | | | |
|  |  | All respondents (n=321) | 57% | cT3 |
|  |  | Radiologists (n=255) | 60% | cT3 |
|  |  | - Abdominal radiologists with specific expertise in rectal MRI (n=103) | 67% | cT3 |
|  |  | - Abdominal radiologists (n=87) | 61% | cT3 |
|  |  | - General radiologists (n=39) | 44% | cT3 |
|  |  | - Senior residents (n=18) | 50% | cT3 |
|  |  | - Junior residents (n=8) | 50% | cT3 |
|  |  | Non-radiologists (n=66) | 49% | cT3 |
|  |  | - Surgeons (n=34) | 41% | cT3 |
|  |  | - Radiation oncologists (n=16) | 81% | cT3 |
|  |  | - Pathologists (n=6) | 33%/33% | cT3/cT4b |
|  |  | - Other (n=10) | 50% | cT4a |
| **Section 2 – Anal sphincter and pelvic floor invasion***  Respondents were asked to assign cT-stage for each case | | | **% consensus** | |
|  | **Case 08: Tumour invading the internal anal sphincter** | | | |
|  |  | All respondents (n=321) | 45% | cT1-2 |
|  |  | Radiologists (n=255) | 46% | cT1-2 |
|  |  | - Abdominal radiologists with specific expertise in rectal MRI (n=103) | 61% | cT1-2 |
|  |  | - Abdominal radiologists (n=87) | 43% | cT3 |
|  |  | - General radiologists (n=39) | 49% | cT3 |
|  |  | - Senior residents (n=18) | 50% | T1-2 |
|  |  | - Junior residents (n=8) | 50% | T3 |
|  |  | Non-radiologists (n=66) | 39% | cT1-2 |
|  |  | - Surgeons (n=34) | 47% | T1-2 |
|  |  | - Radiation oncologists (n=16) | 50% | T3 |
|  |  | - Pathologists (n=6) | 67% | T1-2 |
|  |  | - Other (n=10) | 50% | T3 |
|  | **Case 09: Tumour invading the intersphincteric plane** | | | |
|  |  | All respondents (n=321) | 68% | cT3 |
|  |  | Radiologists (n=255) | 70% | cT3 |
|  |  | - Abdominal radiologists with specific expertise in rectal MRI (n=103) | 77% | cT3 |
|  |  | - Abdominal radiologists (n=87) | 69% | cT3 |
|  |  | - General radiologists (n=39) | 56% | cT3 |
|  |  | - Senior residents (n=18) | 72% | cT3 |
|  |  | - Junior residents (n=8) | 63% | cT3 |
|  |  | Non-radiologists (n=66) | 61% | cT3 |
|  |  | - Surgeons (n=34) | 62% | cT3 |
|  |  | - Radiation oncologists (n=16) | 63% | cT3 |
|  |  | - Pathologists (n=6) | 67% | cT3 |
|  |  | - Other (n=10) | 50% | cT3 |
|  | **Case 10: Tumour invading the external anal sphincter** | | | |
|  |  | All respondents (n=321) | 51% | cT4b |
|  |  | Radiologists (n=255) | 51% | cT4b |
|  |  | - Abdominal radiologists with specific expertise in rectal MRI (n=103) | 59% | cT4b |
|  |  | - Abdominal radiologists (n=87) | 53% | cT4b |
|  |  | - General radiologists (n=39) | 33% | cT4b |
|  |  | - Senior residents (n=18) | 39% | cT4b |
|  |  | - Junior residents (n=8) | 50% | cT4b |
|  |  | Non-radiologists (n=66) | 53% | cT4b |
|  |  | - Surgeons (n=34) | 62% | cT4b |
|  |  | - Radiation oncologists (n=16) | 44% | cT4b |
|  |  | - Pathologists (n=6) | 33% | cT2 |
|  |  | - Other (n=10) | 60% | cT4b |
|  | **Case 11: Tumour invading the pelvic floor (levator ani)** | | | |
|  |  | All respondents (n=321) | 73% | cT4b |
|  |  | Radiologists (n=255) | 74% | cT4b |
|  |  | - Abdominal radiologists with specific expertise in rectal MRI (n=103) | 85% | cT4b |
|  |  | - Abdominal radiologists (n=87) | 68% | cT4b |
|  |  | - General radiologists (n=39) | 62% | cT4b |
|  |  | - Senior residents (n=18) | 72% | cT4b |
|  |  | - Junior residents (n=8) | 75% | cT4b |
|  |  | Non-radiologists (n=66) | 67% | cT4b |
|  |  | - Surgeons (n=34) | 77% | cT4b |
|  |  | - Radiation oncologists (n=16) | 56% | cT4b |
|  |  | - Pathologists (n=6) | 50% | cT4b |
|  |  | - Other (n=10) | 60% | cT4b |
| **Section 3 – Mesorectal Fascia (MRF) involvement**  Respondents were asked to determine for each case whether the MRF is involved (MRF+) or not involved (MRF-) | | | **% consensus** | |
|  | **Case 12: Tumour extending into perirectal fat (below peritoneal reflection), distance of 0 mm between tumour and MRF (i.e., MRF+)** | | | |
|  |  | All respondents (n=321) | 96% | MRF+ |
|  |  | Radiologists (n=255) | 97% | MRF+ |
|  |  | - Abdominal radiologists with specific expertise in rectal MRI (n=103) | 96% | MRF+ |
|  |  | - Abdominal radiologists (n=87) | 99% | MRF+ |
|  |  | - General radiologists (n=39) | 95% | MRF+ |
|  |  | - Senior residents (n=18) | 100% | MRF+ |
|  |  | - Junior residents (n=8) | 100% | MRF+ |
|  |  | Non-radiologists (n=66) | 92% | MRF+ |
|  |  | - Surgeons (n=34) | 94% | MRF+ |
|  |  | - Radiation oncologists (n=16) | 94% | MRF+ |
|  |  | - Pathologists (n=6) | 83% | MRF+ |
|  |  | - Other (n=10) | 90% | MRF+ |
|  | **Case 13: Tumour extending into perirectal fat (below peritoneal reflection), distance of <1 mm between tumour and MRF (i.e., MRF+)** | | | |
|  |  | All respondents (n=321) | 79% | MRF+ |
|  |  | Radiologists (n=255) | 85% | MRF+ |
|  |  | - Abdominal radiologists with specific expertise in rectal MRI (n=103) | 89% | MRF+ |
|  |  | - Abdominal radiologists (n=87) | 79% | MRF+ |
|  |  | - General radiologists (n=39) | 82% | MRF+ |
|  |  | - Senior residents (n=18) | 89% | MRF+ |
|  |  | - Junior residents (n=8) | 88% | MRF+ |
|  |  | Non-radiologists (n=66) | 59% | MRF+ |
|  |  | - Surgeons (n=34) | 71% | MRF+ |
|  |  | - Radiation oncologists (n=16) | 56% | MRF+ |
|  |  | - Pathologists (n=6) | 50% | I do not know |
|  |  | - Other (n=10) | 50% | MRF+/MRF- |
|  | **Case 14: Tumour extending into perirectal fat (below peritoneal reflection), distance of 1-2 mm between tumour and MRF** | | | |
|  |  | All respondents (n=321) | 79% | MRF- |
|  |  | Radiologists (n=255) | 78% | MRF- |
|  |  | - Abdominal radiologists with specific expertise in rectal MRI (n=103) | 72% | MRF- |
|  |  | - Abdominal radiologists (n=87) | 85% | MRF- |
|  |  | - General radiologists (n=39) | 80% | MRF- |
|  |  | - Senior residents (n=18) | 72% | MRF- |
|  |  | - Junior residents (n=8) | 88% | MRF- |
|  |  | Non-radiologists (n=66) | 83% | MRF- |
|  |  | - Surgeons (n=34) | 88% | MRF- |
|  |  | - Radiation oncologists (n=16) | 81% | MRF- |
|  |  | - Pathologists (n=6) | 67% | MRF- |
|  |  | - Other (n=10) | 80% | MRF- |
|  | **Case 15: Tumour extending into perirectal fat anteriorly (above peritoneal reflection), invading the peritoneum (i.e., MRF-)** | | | |
|  |  | All respondents (n=321) | 51% | MRF- |
|  |  | Radiologists (n=255) | 53% | MRF- |
|  |  | - Abdominal radiologists with specific expertise in rectal MRI (n=103) | 61% | MRF- |
|  |  | - Abdominal radiologists (n=87) | 52% | MRF- |
|  |  | - General radiologists (n=39) | 51% | MRF+ |
|  |  | - Senior residents (n=18) | 50% | MRF+ |
|  |  | - Junior residents (n=8) | 50% | MRF- |
|  |  | Non-radiologists (n=66) | 49% | MRF+ |
|  |  | - Surgeons (n=34) | 50% | MRF+ |
|  |  | - Radiation oncologists (n=16) | 69% | MRF+ |
|  |  | - Pathologists (n=6) | 100% | MRF- |
|  |  | - Other (n=10) | 60% | MRF- |
|  | **Case 16: Tumour extending into perirectal fat posteriorly (above peritoneal reflection), distance of 0 mm between tumour and MRF (i.e., MRF+)** | | | |
|  |  | All respondents (n=321) | 86% | MRF+ |
|  |  | Radiologists (n=255) | 85% | MRF+ |
|  |  | - Abdominal radiologists with specific expertise in rectal MRI (n=103) | 89% | MRF+ |
|  |  | - Abdominal radiologists (n=87) | 86% | MRF+ |
|  |  | - General radiologists (n=39) | 72% | MRF+ |
|  |  | - Senior residents (n=18) | 78% | MRF+ |
|  |  | - Junior residents (n=8) | 88% | MRF+ |
|  |  | Non-radiologists (n=66) | 92% | MRF+ |
|  |  | - Surgeons (n=34) | 97% | MRF+ |
|  |  | - Radiation oncologists (n=16) | 94% | MRF+ |
|  |  | - Pathologists (n=6) | 67% | MRF+ |
|  |  | - Other (n=10) | 90% | MRF+ |
|  | **Case 17: N+ Lymph node without extracapsular extension directly adjacent to MRF** | | | |
|  |  | All respondents (n=321) | 57% | MRF- |
|  |  | Radiologists (n=255) | 55% | MRF- |
|  |  | - Abdominal radiologists with specific expertise in rectal MRI (n=103) | 59% | MRF- |
|  |  | - Abdominal radiologists (n=87) | 52% | MRF- |
|  |  | - General radiologists (n=39) | 62% | MRF- |
|  |  | - Senior residents (n=18) | 44% | MRF+ |
|  |  | - Junior residents (n=8) | 75% | MRF- |
|  |  | Non-radiologists (n=66) | 67% | MRF- |
|  |  | - Surgeons (n=34) | 62% | MRF- |
|  |  | - Radiation oncologists (n=16) | 75% | MRF- |
|  |  | - Pathologists (n=6) | 83% | MRF- |
|  |  | - Other (n=10) | 60% | MRF- |
|  | **Case 18: N+ lymph node with extracapsular extension directly adjacent to MRF** | | | |
|  |  | All respondents (n=321) | 85% | MRF+ |
|  |  | Radiologists (n=255) | 87% | MRF+ |
|  |  | - Abdominal radiologists with specific expertise in rectal MRI (n=103) | 89% | MRF+ |
|  |  | - Abdominal radiologists (n=87) | 90% | MRF+ |
|  |  | - General radiologists (n=39) | 72% | MRF+ |
|  |  | - Senior residents (n=18) | 94% | MRF+ |
|  |  | - Junior residents (n=8) | 88% | MRF+ |
|  |  | Non-radiologists (n=66) | 79% | MRF+ |
|  |  | - Surgeons (n=34) | 85% | MRF+ |
|  |  | - Radiation oncologists (n=16) | 81% | MRF+ |
|  |  | - Pathologists (n=6) | 50% | I do not know |
|  |  | - Other (n=10) | 90% | MRF+ |
| **Section 4 – Nodal staging**  For case 17-19 respondents were asked to classify each shown lesion as a node or deposit   For case 20- respondents were asked to assign cN-stage (cN1a, cN1b, cN1c, cN2a, cN2b) for each case | | | **% consensus** | |
|  | **Case 19: Nodular lesion in mesorectum** | | | |
|  |  | All respondents (n=321) | 89% | node |
|  |  | Radiologists (n=255) | 91% | node |
|  |  | - Abdominal radiologists with specific expertise in rectal MRI (n=103) | 94% | node |
|  |  | - Abdominal radiologists (n=87) | 87% | node |
|  |  | - General radiologists (n=39) | 87% | node |
|  |  | - Senior residents (n=18) | 94% | node |
|  |  | - Junior residents (n=8) | 88% | node |
|  |  | Non-radiologists (n=66) | 85% | node |
|  |  | - Surgeons (n=34) | 79% | node |
|  |  | - Radiation oncologists (n=16) | 94% | node |
|  |  | - Pathologists (n=6) | 83% | node |
|  |  | - Other (n=10) | 90% | node |
|  | **Case 20: Irregular mass in mesorectum** | |  |  |
|  |  | All respondents (n=321) | 84% | deposit |
|  |  | Radiologists (n=255) | 84% | deposit |
|  |  | - Abdominal radiologists with specific expertise in rectal MRI (n=103) | 87% | deposit |
|  |  | - Abdominal radiologists (n=87) | 86% | deposit |
|  |  | - General radiologists (n=39) | 77% | deposit |
|  |  | - Senior residents (n=18) | 78% | deposit |
|  |  | - Junior residents (n=8) | 75% | deposit |
|  |  | Non-radiologists (n=66) | 80% | deposit |
|  |  | - Surgeons (n=34) | 82% | deposit |
|  |  | - Radiation oncologists (n=16) | 88% | deposit |
|  |  | - Pathologists (n=6) | 67% | deposit |
|  |  | - Other (n=10) | 70% | deposit |
|  | **Case 21: Partly nodular, partly irregular mass in mesorectum** | | | |
|  |  | All respondents (n=321) | 43% | deposit |
|  |  | Radiologists (n=255) | 46% | deposit |
|  |  | - Abdominal radiologists with specific expertise in rectal MRI (n=103) | 52% | deposit |
|  |  | - Abdominal radiologists (n=87) | 51% | deposit |
|  |  | - General radiologists (n=39) | 39% | node |
|  |  | - Senior residents (n=18) | 44% | node |
|  |  | - Junior residents (n=8) | 75% | node |
|  |  | Non-radiologists (n=66) | 55% | node |
|  |  | - Surgeons (n=34) | 50% | node |
|  |  | - Radiation oncologists (n=16) | 75% | node |
|  |  | - Pathologists (n=6) | 50% | node |
|  |  | - Other (n=10) | 40%/40% | Node/I do not know |
|  | **Case 22: Single metastatic node in mesorectum (i.e., cN1a)** | | | |
|  |  | All respondents (n=321) | 98% | cN1a |
|  |  | Radiologists (n=255) | 98% | cN1a |
|  |  | - Abdominal radiologists with specific expertise in rectal MRI (n=103) | 98% | cN1a |
|  |  | - Abdominal radiologists (n=87) | 98% | cN1a |
|  |  | - General radiologists (n=39) | 95% | cN1a |
|  |  | - Senior residents (n=18) | 100% | cN1a |
|  |  | - Junior residents (n=8) | 100% | cN1a |
|  |  | Non-radiologists (n=66) | 99% | cN1a |
|  |  | - Surgeons (n=34) | 97% | cN1a |
|  |  | - Radiation oncologists (n=16) | 100% | cN1a |
|  |  | - Pathologists (n=6) | 100% | cN1a |
|  |  | - Other (n=10) | 100% | cN1a |
|  | **Case 23: Two metastatic nodes in mesorectum (i.e., cN1b)** | | | |
|  |  | All respondents (n=321) | 94% | cN1b |
|  |  | Radiologists (n=255) | 94% | cN1b |
|  |  | - Abdominal radiologists with specific expertise in rectal MRI (n=103) | 92% | cN1b |
|  |  | - Abdominal radiologists (n=87) | 93% | cN1b |
|  |  | - General radiologists (n=39) | 97% | cN1b |
|  |  | - Senior residents (n=18) | 100% | cN1b |
|  |  | - Junior residents (n=8) | 100% | cN1b |
|  |  | Non-radiologists (n=66) | 96% | cN1b |
|  |  | - Surgeons (n=34) | 91% | cN1b |
|  |  | - Radiation oncologists (n=16) | 100% | cN1b |
|  |  | - Pathologists (n=6) | 100% | cN1b |
|  |  | - Other (n=10) | 100% | cN1b |
| **Case 24: Single tumour deposit in mesorectum with no additional suspicious nodes (i.e. cN1c)** | | | | |
|  |  | All respondents (n=321) | 92% | cN1c |
|  |  | Radiologists (n=255) | 92% | cN1c |
|  |  | - Abdominal radiologists with specific expertise in rectal MRI (n=103) | 93% | cN1c |
|  |  | - Abdominal radiologists (n=87) | 92% | cN1c |
|  |  | - General radiologists (n=39) | 87% | cN1c |
|  |  | - Senior residents (n=18) | 89% | cN1c |
|  |  | - Junior residents (n=8) | 100% | cN1c |
|  |  | Non-radiologists (n=66) | 91% | cN1c |
|  |  | - Surgeons (n=34) | 91% | cN1c |
|  |  | - Radiation oncologists (n=16) | 81% | cN1c |
|  |  | - Pathologists (n=6) | 100% | cN1c |
|  |  | - Other (n=10) | 100% | cN1c |
|  | **Case 25: Single tumour deposit plus single metastatic node in mesorectum** | | | |
|  |  | All respondents (n=321) | 52% | cN1c |
|  |  | Radiologists (n=255) | 54% | cN1c |
|  |  | - Abdominal radiologists with specific expertise in rectal MRI (n=103) | 53% | cN1c |
|  |  | - Abdominal radiologists (n=87) | 54% | cN1c |
|  |  | - General radiologists (n=39) | 49% | cN1c |
|  |  | - Senior residents (n=18) | 56% | cN1c |
|  |  | - Junior residents (n=8) | 88% | cN1c |
|  |  | Non-radiologists (n=66) | 46% | cN1c |
|  |  | - Surgeons (n=34) | 47% | cN1c |
|  |  | - Radiation oncologists (n=16) | 50% | cN1c |
|  |  | - Pathologists (n=6) | 50% | cN1a |
|  |  | - Other (n=10) | 60% | cN1c |
|  | **Case 26: Seven metastatic lymph nodes in mesorectum (i.e., cN2b)** | | | |
|  |  | All respondents (n=321) | 95% | cN2b |
|  |  | Radiologists (n=255) | 96% | cN2b |
|  |  | - Abdominal radiologists with specific expertise in rectal MRI (n=103) | 97% | cN2b |
|  |  | - Abdominal radiologists (n=87) | 99% | cN2b |
|  |  | - General radiologists (n=39) | 90% | cN2b |
|  |  | - Senior residents (n=18) | 94% | cN2b |
|  |  | - Junior residents (n=8) | 88% | cN2b |
|  |  | Non-radiologists (n=66) | 91% | cN2b |
|  |  | - Surgeons (n=34) | 85% | cN2b |
|  |  | - Radiation oncologists (n=16) | 100% | cN2b |
|  |  | - Pathologists (n=6) | 100% | cN2b |
|  |  | - Other (n=10) | 90% | cN2b |
|  | **Case 27: Four metastatic lymph nodes in mesorectum (i.e., cN2a)** | | | |
|  |  | All respondents (n=321) | 94% | cN2a |
|  |  | Radiologists (n=255) | 94% | cN2a |
|  |  | - Abdominal radiologists with specific expertise in rectal MRI (n=103) | 96% | cN2a |
|  |  | - Abdominal radiologists (n=87) | 97% | cN2a |
|  |  | - General radiologists (n=39) | 85% | cN2a |
|  |  | - Senior residents (n=18) | 100% | cN2a |
|  |  | - Junior residents (n=8) | 75% | cN2a |
|  |  | Non-radiologists (n=66) | 96% | cN2a |
|  |  | - Surgeons (n=34) | 94% | cN2a |
|  |  | - Radiation oncologists (n=16) | 94% | cN2a |
|  |  | - Pathologists (n=6) | 100% | cN2a |
|  |  | - Other (n=10) | 100% | cN2a |
| **Section 5 – Regional versus non-regional lymph nodes**  Respondents were asked to determine whether nodes were regional (N) or non-regional (M) lymph nodes | | | **% consensus** | |
|  | **Case 28: Mesorectal lymph node (i.e., regional)** | | | |
|  |  | All respondents (n=321) | 100% | regional |
|  |  | Radiologists (n=255) | 100% | regional |
|  |  | - Abdominal radiologists with specific expertise in rectal MRI (n=103) | 100% | regional |
|  |  | - Abdominal radiologists (n=87) | 100% | regional |
|  |  | - General radiologists (n=39) | 100% | regional |
|  |  | - Senior residents (n=18) | 100% | regional |
|  |  | - Junior residents (n=8) | 100% | regional |
|  |  | Non-radiologists (n=66) | 100% | regional |
|  |  | - Surgeons (n=34) | 100% | regional |
|  |  | - Radiation oncologists (n=16) | 100% | regional |
|  |  | - Pathologists (n=6) | 100% | regional |
|  |  | - Other (n=10) | 100% | regional |
|  | **Case 29: Obturator lymph node (i.e., regional)** | | | |
|  |  | All respondents (n=321) | 58% | regional |
|  |  | Radiologists (n=255) | 55% | regional |
|  |  | - Abdominal radiologists with specific expertise in rectal MRI (n=103) | 58% | regional |
|  |  | - Abdominal radiologists (n=87) | 51% | regional |
|  |  | - General radiologists (n=39) | 54% | regional |
|  |  | - Senior residents (n=18) | 61% | regional |
|  |  | - Junior residents (n=8) | 63% | regional |
|  |  | Non-radiologists (n=66) | 67% | regional |
|  |  | - Surgeons (n=34) | 62% | regional |
|  |  | - Radiation oncologists (n=16) | 88% | regional |
|  |  | - Pathologists (n=6) | 67% | regional |
|  |  | - Other (n=10) | 50%/50% | Regional/non-regional |
|  | **Case 30: External iliac lymph node (i.e., non-regional)** | | | |
|  |  | All respondents (n=321) | 80% | non-regional |
|  |  | Radiologists (n=255) | 83% | non-regional |
|  |  | - Abdominal radiologists with specific expertise in rectal MRI (n=103) | 88% | non-regional |
|  |  | - Abdominal radiologists (n=87) | 83% | non-regional |
|  |  | - General radiologists (n=39) | 77% | non-regional |
|  |  | - Senior residents (n=18) | 61% | non-regional |
|  |  | - Junior residents (n=8) | 88% | non-regional |
|  |  | Non-radiologists (n=66) | 71% | non-regional |
|  |  | - Surgeons (n=34) | 79% | non-regional |
|  |  | - Radiation oncologists (n=16) | 56% | non-regional |
|  |  | - Pathologists (n=6) | 50% | non-regional |
|  |  | - Other (n=10) | 80% | non-regional |
|  | **Case 31: Internal iliac lymph node (i.e., regional)** | | | |
|  |  | All respondents (n=321) | 67% | regional |
|  |  | Radiologists (n=255) | 67% | regional |
|  |  | - Abdominal radiologists with specific expertise in rectal MRI (n=103) | 65% | regional |
|  |  | - Abdominal radiologists (n=87) | 66% | regional |
|  |  | - General radiologists (n=39) | 62% | regional |
|  |  | - Senior residents (n=18) | 89% | regional |
|  |  | - Junior residents (n=8) | 88% | regional |
|  |  | Non-radiologists (n=66) | 68% | regional |
|  |  | - Surgeons (n=34) | 59% | regional |
|  |  | - Radiation oncologists (n=16) | 88% | regional |
|  |  | - Pathologists (n=6) | 67% | regional |
|  |  | - Other (n=10) | 70% | regional |
|  | **Case 32: Common iliac lymph node (i.e. non-regional)** | | | |
|  |  | All respondents (n=321) | 85% | non-regional |
|  |  | Radiologists (n=255) | 87% | non-regional |
|  |  | - Abdominal radiologists with specific expertise in rectal MRI (n=103) | 90% | non-regional |
|  |  | - Abdominal radiologists (n=87) | 84% | non-regional |
|  |  | - General radiologists (n=39) | 82% | non-regional |
|  |  | - Senior residents (n=18) | 89% | non-regional |
|  |  | - Junior residents (n=8) | 88% | non-regional |
|  |  | Non-radiologists (n=66) | 79% | non-regional |
|  |  | - Surgeons (n=34) | 79% | non-regional |
|  |  | - Radiation oncologists (n=16) | 81% | non-regional |
|  |  | - Pathologists (n=6) | 67% | non-regional |
|  |  | - Other (n=10) | 80% | non-regional |
|  | **Case 33: Inguinal node in distal tumour extending below dentate line (i.e., regional)** | | | |
|  |  | All respondents (n=321) | 51% | non-regional |
|  |  | Radiologists (n=255) | 51% | non-regional |
|  |  | - Abdominal radiologists with specific expertise in rectal MRI (n=103) | 48% | non-regional |
|  |  | - Abdominal radiologists (n=87) | 59% | non-regional |
|  |  | - General radiologists (n=39) | 49% | regional |
|  |  | - Senior residents (n=18) | 56% | regional |
|  |  | - Junior residents (n=8) | 75% | non-regional |
|  |  | Non-radiologists (n=66) | 47% | non-regional |
|  |  | - Surgeons (n=34) | 47%/47% | regional/non-regional |
|  |  | - Radiation oncologists (n=16) | 56% | regional |
|  |  | - Pathologists (n=6) | 50% | non-regional |
|  |  | - Other (n=10) | 60% | non-regional |
|  | **Case 34: Inguinal node in mid-rectal tumour not extending into the anal canal (i.e., non-regional)** | | | |
|  |  | All respondents (n=321) | 96% | non-regional |
|  |  | Radiologists (n=255) | 95% | non-regional |
|  |  | - Abdominal radiologists with specific expertise in rectal MRI (n=103) | 97% | non-regional |
|  |  | - Abdominal radiologists (n=87) | 95% | non-regional |
|  |  | - General radiologists (n=39) | 95% | non-regional |
|  |  | - Senior residents (n=18) | 95% | non-regional |
|  |  | - Junior residents (n=8) | 75% | non-regional |
|  |  | Non-radiologists (n=66) | 99% | non-regional |
|  |  | - Surgeons (n=34) | 100% | non-regional |
|  |  | - Radiation oncologists (n=16) | 94% | non-regional |
|  |  | - Pathologists (n=6) | 100% | non-regional |
|  |  | - Other (n=10) | 100% | non-regional |
| **Section 6 – M-staging**  Respondents were asked to assign cM-stage (cM1a, cM1b, cM1c) | | | **% consensus** | |
|  | **Case 35: Common iliac lymph node metastasis (i.e. cM1a)** | | | |
|  |  | All respondents (n=321) | 94% | cM1a |
|  |  | Radiologists (n=255) | 93% | cM1a |
|  |  | - Abdominal radiologists with specific expertise in rectal MRI (n=103) | 91% | cM1a |
|  |  | - Abdominal radiologists (n=87) | 97% | cM1a |
|  |  | - General radiologists (n=39) | 92% | cM1a |
|  |  | - Senior residents (n=18) | 89% | cM1a |
|  |  | - Junior residents (n=8) | 100% | cM1a |
|  |  | Non-radiologists (n=66) | 97% | cM1a |
|  |  | - Surgeons (n=34) | 97% | cM1a |
|  |  | - Radiation oncologists (n=16) | 100% | cM1a |
|  |  | - Pathologists (n=6) | 83% | cM1a |
|  |  | - Other (n=10) | 100% | cM1a |
|  | **Case 36: Liver + para-aortic lymph node metastases (i.e., cM1b)** | | | |
|  |  | All respondents (n=321) | 94% | cM1b |
|  |  | Radiologists (n=255) | 93% | cM1b |
|  |  | - Abdominal radiologists with specific expertise in rectal MRI (n=103) | 94% | cM1b |
|  |  | - Abdominal radiologists (n=87) | 90% | cM1b |
|  |  | - General radiologists (n=39) | 92% | cM1b |
|  |  | - Senior residents (n=18) | 100% | cM1b |
|  |  | - Junior residents (n=8) | 100% | cM1b |
|  |  | Non-radiologists (n=66) | 96% | cM1b |
|  |  | - Surgeons (n=34) | 94% | cM1b |
|  |  | - Radiation oncologists (n=16) | 100% | cM1b |
|  |  | - Pathologists (n=6) | 100% | cM1b |
|  |  | - Other (n=10) | 90% | cM1b |
|  | **Case 37: Unilateral lung metastases (right lung) (i.e. M1a)** | | | |
|  |  | All respondents (n=321) | 84% | cM1a |
|  |  | Radiologists (n=255) | 83% | cM1a |
|  |  | - Abdominal radiologists with specific expertise in rectal MRI (n=103) | 90% | cM1a |
|  |  | - Abdominal radiologists (n=87) | 79% | cM1a |
|  |  | - General radiologists (n=39) | 72% | cM1a |
|  |  | - Senior residents (n=18) | 89% | cM1a |
|  |  | - Junior residents (n=8) | 75% | cM1a |
|  |  | Non-radiologists (n=66) | 86% | cM1a |
|  |  | - Surgeons (n=34) | 88% | cM1a |
|  |  | - Radiation oncologists (n=16) | 75% | cM1a |
|  |  | - Pathologists (n=6) | 100% | cM1a |
|  |  | - Other (n=10) | 90% | cM1a |
|  | **Case 38: Bilateral lung metastases (right + left lung) (i.e. M1a)** | | | |
|  |  | All respondents (n=321) | 56% | cM1b |
|  |  | Radiologists (n=255) | 58% | cM1b |
|  |  | - Abdominal radiologists with specific expertise in rectal MRI (n=103) | 51% | cM1b |
|  |  | - Abdominal radiologists (n=87) | 63% | cM1b |
|  |  | - General radiologists (n=39) | 56% | cM1b |
|  |  | - Senior residents (n=18) | 67% | cM1b |
|  |  | - Junior residents (n=8) | 75% | cM1b |
|  |  | Non-radiologists (n=66) | 50% | cM1b |
|  |  | - Surgeons (n=34) | 41%/41% | cM1a/cM1b |
|  |  | - Radiation oncologists (n=16) | 50%/50% | cM1a/cM1b |
|  |  | - Pathologists (n=6) | 100% | cM1b |
|  |  | - Other (n=10) | 50% | cM1b |
|  | **Case 39: Liver + renal + spleen metastases (i.e. M1b)** | | | |
|  |  | All respondents (n=321) | 86% | cM1b |
|  |  | Radiologists (n=255) | 85% | cM1b |
|  |  | - Abdominal radiologists with specific expertise in rectal MRI (n=103) | 88% | cM1b |
|  |  | - Abdominal radiologists (n=87) | 83% | cM1b |
|  |  | - General radiologists (n=39) | 80% | cM1b |
|  |  | - Senior residents (n=18) | 83% | cM1b |
|  |  | - Junior residents (n=8) | 100% | cM1b |
|  |  | Non-radiologists (n=66) | 88% | cM1b |
|  |  | - Surgeons (n=34) | 82% | cM1b |
|  |  | - Radiation oncologists (n=16) | 100% | cM1b |
|  |  | - Pathologists (n=6) | 100% | cM1b |
|  |  | - Other (n=10) | 80% | cM1b |
|  | **Case 40: Peritoneal metastases (i.e. M1c)** | | | |
|  |  | All respondents (n=321) | 97% | cM1c |
|  |  | Radiologists (n=255) | 97% | cM1c |
|  |  | - Abdominal radiologists with specific expertise in rectal MRI (n=103) | 96% | cM1c |
|  |  | - Abdominal radiologists (n=87) | 99% | cM1c |
|  |  | - General radiologists (n=39) | 95% | cM1c |
|  |  | - Senior residents (n=18) | 100% | cM1c |
|  |  | - Junior residents (n=8) | 88% | cM1c |
|  |  | Non-radiologists (n=66) | 97% | cM1c |
|  |  | - Surgeons (n=34) | 97% | cM1c |
|  |  | - Radiation oncologists (n=16) | 94% | cM1c |
|  |  | - Pathologists (n=6) | 100% | cM1c |
|  |  | - Other (n=10) | 100% | cM1c |
|  | **Case 41: Peritoneal + liver metastases (i.e., cM1c)** | | | |
|  |  | All respondents (n=321) | 97% | cM1c |
|  |  | Radiologists (n=255) | 97% | cM1c |
|  |  | - Abdominal radiologists with specific expertise in rectal MRI (n=103) | 95% | cM1c |
|  |  | - Abdominal radiologists (n=87) | 99% | cM1c |
|  |  | - General radiologists (n=39) | 97% | cM1c |
|  |  | - Senior residents (n=18) | 100% | cM1c |
|  |  | - Junior residents (n=8) | 88% | cM1c |
|  |  | Non-radiologists (n=66) | 99% | cM1c |
|  |  | - Surgeons (n=34) | 97% | cM1c |
|  |  | - Radiation oncologists (n=16) | 100% | cM1c |
|  |  | - Pathologists (n=6) | 100% | cM1c |
|  |  | - Other (n=10) | 100% | cM1c |
| Note, the “Other” respondents included 7 medical oncologists, 2 gastroenterologists and 1 PhD student.  * in cases related to cT-staging, the answer options cT1, cT2 and cT12 (unable to differentiate between cT1 and cT2) were grouped together for calculation of agreement. In all other cases agreement was calculated based on individual answer options. | | | | |
